# Insect‐Associated Bacteria Assemble the Antifungal Butenolide Gladiofungin by Non‐Canonical Polyketide Chain Termination

**DOI:** 10.1002/anie.202005711

**Published:** 2020-10-15

**Authors:** Sarah P. Niehs, Jana Kumpfmüller, Benjamin Dose, Rory F. Little, Keishi Ishida, Laura V. Flórez, Martin Kaltenpoth, Christian Hertweck

**Affiliations:** ^1^ Department of Biomolecular Chemistry Leibniz Institute for Natural Product Research and Infection Biology, HKI Beutenbergstr. 11a 07745 Jena Germany; ^2^ Department for Evolutionary Ecology Institute of Organismic and Molecular Evolution Johannes Gutenberg University Mainz Hanns-Dieter-Hüsch-Weg 15 55128 Mainz Germany; ^3^ Faculty of Biological Sciences Friedrich Schiller University Jena 07743 Jena Germany

**Keywords:** antifungal compounds, biosynthesis, genome mining, natural products, polyketides

## Abstract

Genome mining of one of the protective symbionts (*Burkholderia gladioli*) of the invasive beetle *Lagria villosa* revealed a cryptic gene cluster that codes for the biosynthesis of a novel antifungal polyketide with a glutarimide pharmacophore. Targeted gene inactivation, metabolic profiling, and bioassays led to the discovery of the gladiofungins as previously‐overlooked components of the antimicrobial armory of the beetle symbiont, which are highly active against the entomopathogenic fungus *Purpureocillium lilacinum*. By mutational analyses, isotope labeling, and computational analyses of the modular polyketide synthase, we found that the rare butenolide moiety of gladiofungins derives from an unprecedented polyketide chain termination reaction involving a glycerol‐derived C3 building block. The key role of an A‐factor synthase (AfsA)‐like offloading domain was corroborated by CRISPR‐Cas‐mediated gene editing, which facilitated precise excision within a PKS domain.

## Graphical abstract

Genome mining, bioactivity and metabolic profiling revealed a novel polyketide (gladiofungin) that augments the antifungal armory of pest beetles’ protective symbionts. Its non‐canonical multimodular assembly line employs an unprecedented polyketide chain termination reaction to form a butenolide ring, as demonstrated by isotope labeling and CRISPR‐Cas gene editing.

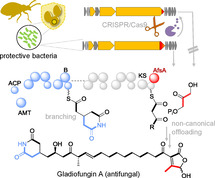

Natural products at the interface of hosts and their associated microbes play important roles as chemical mediators that have been optimized for their particular functions. In addition to their specific ecological roles, these molecules may contribute to the development of pharmaceuticals.^[1]^ In particular, protective symbioses, where microbes confer an evolutionary benefit to the host by warding off pathogens, grazers or competitors, are a rich source of cytotoxins and antimicrobials. Important examples include endophytes,^[2]^ symbionts of marine invertebrates,^[3]^ and bacteria associated to nematodes and insects.^[4]^ Females of the invasive beetle *Lagria villosa* protect their offspring from environmental competitors by applying a bacteria‐containing secretion onto the eggs.^[5]^ The associated bacteria (*Burkholderia* spp.) secrete a broad spectrum of antimicrobial agents and may thus prevent infections by entomopathogenic bacteria and fungi (Figure 1 A).[^5b^, ^5c^] By chemical analyses of bacterial cultures and egg clutches we have discovered and characterized a range of toxins and antimicrobials produced by culturable and non‐culturable *Burkholderia* symbionts.[^5b^, ^6^] Among these, we identified the complex polyketide lagriamide, the polyyne caryoynencin, and the isothiocyanate sinapigladioside as potent antifungal agents against the entomopathogen *Purpureocillium lilacinum*, likely one of the major environmental rivals of the beetle.[^5b^, ^5c^] Genome analyses, however, indicate an even higher biosynthetic potential of the bacterial symbionts.^[7]^ Apparently, the full chemical armory that is used to maintain and stabilize the *Lagria‐Burkholderia* symbioses is still unknown. Here, we report the genomics‐driven discovery of a symbiont‐derived, antifungal agent with an unusual butenolide residue that results from a non‐canonical polyketide chain‐termination reaction. Our study presents a new polyketide synthase (PKS) offloading mechanism that is an important addition to the current PKS toolbox and may inspire synthetic biology approaches to increase structural diversity.

**Figure 1:**
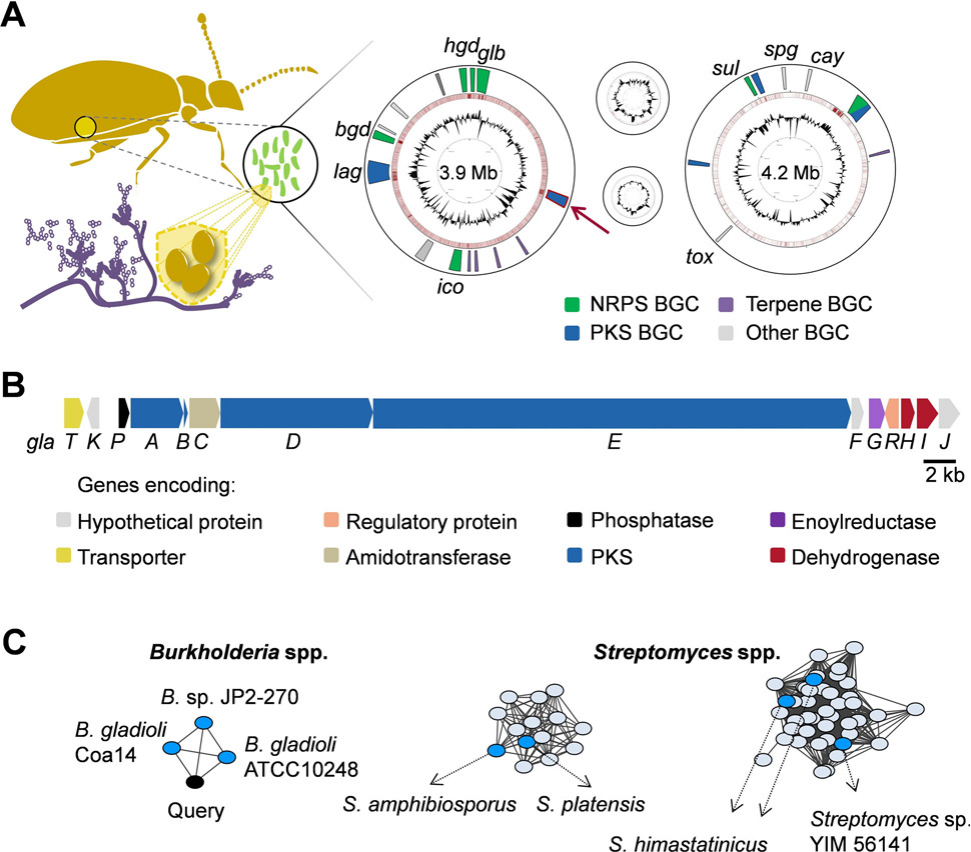
Protective *Lagria‐Burkholderia* symbiosis. A) Biosynthetic potential encoded in the genome of the symbiont *Burkholderia gladioli* HKI0739. Chromosomes: inner circle represents GC content; the second circle shows known gene products (red); and the third circle shows identified BGCs. *Bgd*=BGC for burriogladin, *hgd*=haereogladin, *glb*=gladiobactin, *ico*=icosalide, *lag*=lagriene, *spg*=sinapigladioside, *cay*=caryoynencin, *sul*=sulfazecin, *tox*=toxoflavin. The cryptic BGC studied is marked with an arrow. B) Cryptic PKS gene cluster *gla*. C) Sequence similarity network created with the deduced amino acid sequence of GlaD as query to the EFI‐EST web tool. Dark blue=known gene cluster, black=query, light blue=orphan gene cluster, *B*. sp.=*Burkholderia* sp.

Mining the genome sequence of beetle symbiont *Burkholderia gladioli* HKI0739 (*syn*. Lv‐StA; Genome accession number: GCA_009912275), we noted an orphan gene cluster (54 kb) that codes for a trans‐AT modular polyketide synthase (GlaABDE; Figure 1 B) and accessory enzymes. The encoded assembly line appeared to be worth investigating because in lieu of the canonical thioesterase (TE) domain for chain release the *gla* PKS features a domain that shares similarity with AfsA, an A‐factor biosynthesis protein (pfam 03756),^[8]^ and thus point to a non‐canonical chain termination mechanism.

For a detailed analysis of the *gla* PKS, the deduced amino acid sequences of GlaABDE were submitted as query to the EFI‐EST (enzyme function initiative: enzyme similarity tool)^[9]^ to create SSNs (sequence similarity networks), and the results were forwarded to the genome neighborhood tool (EFI‐GNT). The SSN using the amino acid sequence of GlaD as query revealed three significant neural networks (Figure 1 C), one SSN of BGCs encoded in genomes of *Burkholderia* spp. and, surprisingly, two prominently BGCs encoded in *Streptomyces* spp. Notably, the assembly lines from *Streptomyces* neural networks are described to code for glutarimide‐substituted compounds.^[10]^ For more details also see Supporting Information and Figure S1.

Manual inspection of the networks showed a high gene synteny of the genes for glutarimide formation (Figure 2 A). A detailed in silico analysis of the module architecture indicated a designated branching module similar to the one studied in the rhizoxin (*rhi*) PKS.^[11]^ The KS, B (sometimes denoted as X), and ACP domains have been implicated for the biosynthesis of the pharmacophoric δ‐lactone and glutarimide moieties that confer cytotoxic and antifungal activities.[^10b^, ^10c^, ^12^] Examination of the deduced assembly line suggested that the *gla* pathway is initiated by the formation of ACP‐bound malonamate, followed by chain elongation and construction of an α,β‐unsaturated diketide intermediate. Then, the branching module would mediate a Michael‐type vinylogous addition of a malonyl unit, followed by cyclization to generate the glutarimide moiety (Figure 2 A).[^10c^, ^13^]

**Figure 2:**
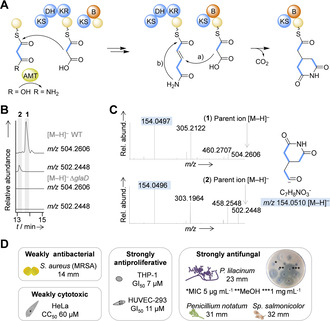
Biosynthetic origin of glutarimides and bioactivity of gladiofungin. A) Glutarimide assembly based on deduced domain architecture. AMT=amidotransferase, KS=ketosynthase. DH=dehydratase, KR=ketoreductase, B=branching, yellow spheres=acyl carrier protein. B) Production of glutarimide‐substituted compounds **1** and **2** in *B. gladioli* HKI0739 (WT) vs. metabolic profile of deletion mutant Δ*glaD*. C) MS/MS fragmentation patterns of **1** and **2** in negative mode showing predicted glutarimide‐containing fragment. D) Antimicrobial properties and cytotoxic activities of **1**. Results of agar diffusion assay in mm with 9 mm hole diameter. *Sp*.=*Sporobolomyces. P*.=*Purpureocillium*. Agar diffusion assays performed with 1 mg mL^−1^ if not stated otherwise.

To discover a glutarimide‐bearing polyketide, we investigated cultures of the beetle symbionts by HR‐ESI‐LC/MS and identified two nitrogen‐bearing compounds that could be the products of the *gla* assembly line: a major metabolite (**1**) of *m*/*z* 504.2606 [*M*−H]^−^ and a minor congener (**2**) of *m*/*z* 502.2448 [*M*−H]^−^ with deduced molecular formulas of C_27_H_38_NO_8_ and C_27_H_36_NO_8_, respectively (Figure 2 B). Both MS/MS fragmentation patterns showed a prominent fragment of 154.0496 [*M*−H]^−^ (calculated chemical formula of C_7_H_8_NO_3_^−^) characteristic for a glutarimide moiety (Figure 2 C).

To unequivocally correlate the *gla* gene cluster with the production of **1** and **2**, we constructed a targeted gene deletion mutant by homologous recombination. Using the knockout plasmid pJK345, a kanamycin resistance cassette was integrated into the PKS gene *glaD*. The correct integration into the symbiont's genome was verified by PCR and sequencing (see Supporting Information). HPLC‐MS analysis of the Δ*glaD* mutant extract showed that the disruption results in a non‐producing strain (Figure 2 B).

To obtain **1** in sufficient amounts for a full characterization, we subjected the crude extract from a scaled up fermentation broth (5.6 L) to fractionations via size‐exclusion chromatography. Subsequent repeated preparative RP‐HPLC yielded 13 mg (2.3 mg L^−1^) of pure **1** (Supporting Information). The minor congener **2** was only produced in minute amounts (90 μg L^−1^). In a panel of whole‐cell bioactivity assays, **1** showed only weak antibacterial effects. In contrast, we observed antiproliferative effects of **1** against THP‐1 (GI_50_ 7.1 μm) and HEK‐293 cells (GI_50_ 11.1 μm), with only low cytotoxicity (CC_50_ HeLa cells 60.6 μm). Furthermore, **1** proved to be a potent antifungal agent that inhibits *Penicillium notatum* and *Sporobolomyces salmonicolor* (Figure 2 D and Supporting Information). Notably, **1** inhibits the growth of the insect pathogen *P. lilacinum* already at 5 μg mL^−1^. Owing to its biological origin and its pronounced antifungal activity, we named compound **1** gladiofungin A and the minor congener **2** gladiofungin B.

By means of in silico analyses including a KS‐based phylogeny, we inferred the polyketide backbone of the encoded metabolite. The pronounced similarity to glutarimide‐forming assembly lines encoded in genomes of several *Streptomyces* spp. (modules 1–5) was also taken into account,^[14]^ for example, indicating a trans‐acting methyltransferase (MT5) in module 4. Yet, owing to the non‐canonical off‐loading the substructure at the carboxy terminus of the polyketide chain could not be predicted (Figure 3 A and Supporting Information).

**Figure 3:**
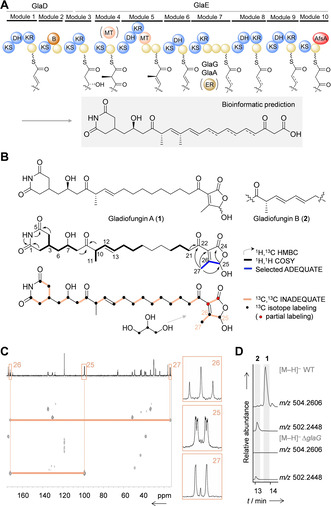
Structure elucidation of gladiofungins. A) Gladiofungin assembly line with predicted KS specificities and putative product. KS=ketosynthase, DH=dehydratase, KR=ketoreductase, B=branching, MT=methyltransferase, AfsA=A‐factor‐biosynthesis domain, yellow spheres=acyl carrier protein. B) Structure elucidation of gladiofungins with 2D NMR couplings and isotope labeling. C) Part of the ^13^C, ^13^C INADEQUATE spectrum of ^13^C‐labeled **1** showing C3 incorporation into butenolide moiety. D) Production of **1** and **2** in *B. gladioli* HKI0739 (WT) vs. metabolic profile of deletion mutant Δ*glaG*.

The structure of **1** was elucidated by 1D‐ and 2D‐NMR. ^13^C NMR and DEPT‐135 data of **1** indicated the presence of six methines, 12 methylenes, two methyl groups, and seven quaternary carbons, from which five were assigned to carbonyls. Based on ^13^C,^1^H HMBC couplings of NH, C‐1 and C‐5 in combination with ^1^H,^1^H‐COSY couplings the glutarimide moiety was deduced. ^1^H,^1^H COSY data revealed that the glutarimide is linked to a carbon chain that is composed of methines, methylenes, and two carbonyl groups (C‐9 and C‐22). Furthermore, we located a carbon‐carbon double bond between CH‐12 and CH‐13 (*δ*_H_ 5.36 and 5.60 ppm). The *E*‐configuration of the double bond was assigned based on the proton coupling constant *J*_12‐13_ 15.5 Hz. MS and NMR data indicated that the carbonyl resonating at *δ*_C_ 199 ppm is part of a moiety that was hard to elucidate. To investigate NMR correlations within this moiety it was necessary to increase ^13^C signal intensity. Upon supplementation of cultures with ^13^C_3_‐labeled glycerol, we observed incorporation of ^13^C‐enriched acetate units into the polyketide backbone as well as an intact C3 unit into the heterocycle (Figures 3 B and C). An ADEQUATE NMR experiment helped identifying neighbor correlation between C‐25, C‐26 and the methyl group at *δ*_H_ 2.24 ppm (H‐27). Furthermore, INADEQUATE data revealed weak 2D coupling between C‐23 and C‐24, which finally established the butenolide substructure of **1**.

Overall, the structure elucidation matches with the in silico prediction of the polyketide part of the molecule (Figures 3 A and B). Since **1** proved to be unstable under the conditions used for Mosher derivatization, we inferred the *R* configuration of the alcohol from the specificity of the KR in module 3 (Figure 3 B).^[15]^ Likewise, we concluded the *S* configuration at C‐10 from the specificity of the KR domain in module 5.^[16]^ Comparisons between ^1^H NMR spectra of **1** and **2** indicate that **2** harbors an additional *E*‐configured double bond between C‐14 and C‐15 (Figure 3 B and Supporting Information). To corroborate the biogenetic relationship of **1** and **2**, we created a knockout by targeting the gene coding for a putative enoyl reductase (GlaG) that would act in trans. We found that **2** is enriched in the Δ*glaG* mutant whereas the production of **1** is abrogated (Figure 3 D).

The structures **1** and **2** are intriguing because they represent a merger of the streptomycetes glutarimide pharmacophore and a butenolide heterocycle. Related butenolide/butyrolactone substructures are found in streptomycetes signaling molecules such as the butyrolactone A‐factor (**3**) and the butenolide SRB (*Streptomyces rochei* butenolide, **4**), which are known to regulate secondary metabolism and morphogenesis.[^8^, ^17^] Owing to the importance of this natural product family, butenolide and related biosynthesis (e.g., **5**) has been studied extensively (Figure 4 A).[^8^, ^18^] In general, C3 precursors such as dihydroxy acetone phosphate (DHAP) are derived from glycolysis and modified by action of AfsA‐like or FkbH‐like proteins. The finding of AfsA‐like domains as components of a multimodular PKS, however, is highly unexpected. By analogy to butenolide biosynthesis, it would be conceivable that the nascent, ACP‐bound polyketide chain would be cleaved by attack of DHAP.

**Figure 4:**
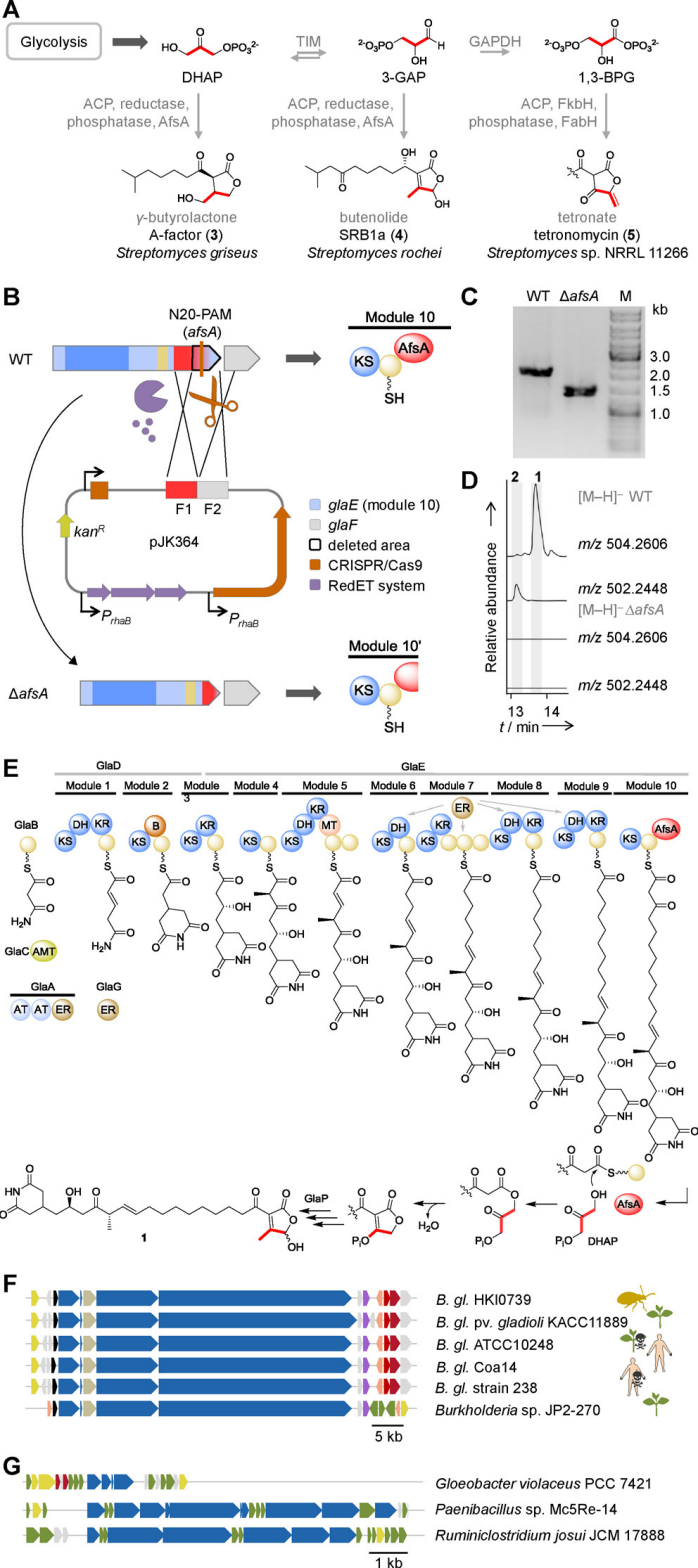
Model of gladiofungin biosynthesis and BGC distribution in other bacterial strains. A) Biosynthetic origin of butenolides, butyrolactones and tetronates in natural products. B) Mutant construction using CRISPR/Cas in *B. gladioli*. C) PCR‐based verification of Δ*afsA* mutant. D) Production of **1** and **2** in *B. gladioli* HKI0739 (WT) vs. metabolic profile of CRISPR/Cas deletion mutant Δ*afsA*. E) Model for gladiofungin biosynthesis and proposed mechanism for butenolide and glutarimide assembly. AMT=amidotransferase, KS=ketosynthase, DH=dehydratase, KR=ketoreductase, B=branching, MT=methyltransferase, ER=enoyl reductase, AT=acyltransferase, AfsA=A‐factor‐biosynthesis domain, yellow spheres, acyl carrier protein. F) Homologous gladiofungin gene clusters in *Burkholderia gladioli* (*B. gl*.) genomes from diverse environmental niches. Colors as in Figure 1 B. G) Selected PKS or PKS/NRPS assembly lines without a dedicated TE but with a terminal AfsA domain. Green=additional genes.

To prove the involvement of the AfsA domain in gladiofungin biosynthesis, we aimed at generating a suitable mutant strain. In particular, we sought to achieve a surgical, marker‐less gene deletion to avoid possible polar effects. Therefore, we established a working CRISPR/Cas gene editing system for *Burkholderia* for the first time (Figure 4 B,C). Specifically, we used a rhamnose‐inducible *Burkholderia*‐optimized *red*‐operon to enhance homologous recombination^[19]^ and a single‐copy, inducible, codon‐optimized *cas9* to cause a double‐strand break at a specific position as determined by the N20‐sequence of the sgRNA. In this way, we were able to precisely target the gene region for the AfsA domain and generated the desired mutant Δ*afsA* (65 % of domain from its center to its C‐terminus was deleted). HPLC analysis revealed that gladiofungin biosynthesis was fully abrogated in this mutant, clearly indicating that the AfsA domain plays a key role in the biosynthetic pathway (Figure 4 D). By sequencing of the three most‐likely regions for off‐target effects we could confirm the specificity of the chosen guide‐RNA (Figure S8). Next to that, we performed a qualitative transcriptome analysis and could not detect significant differences concerning the expression of the downstream genes *glaF* and *glaG* (Figure S11). We also generated a point mutation mutant using the same guide‐RNA (GlaE E10080A). Since the metabolic profile of this mutant is equivalent to the wild type strain (Figure S9), we could irrevocably show that the *glaE* gene was specifically edited without losing PKS functionality and without causing polar effects as well as crucial off‐target effects.

As a surrogate of a canonical, off‐loading TE domain, the AfsA domain would liberate the polyketide chain by attachment of DHAP (Figure 4 E). The butenolide ring formation would involve and intramolecular aldol (Knoevenagel) condensation and dephosphorylation. A possible candidate for the dephosphorylation step is the deduced gene product of *glaP*, which shares similarities to phosphatases (Table S2).

It is remarkable that the *gla* gene cluster is highly conserved in diverse *B. gladioli* strains. We detected 11 orthologous gene clusters in the sequenced genomes of *B. gladioli* pathovars including mushroom, plant and human pathogens (Figure 4 F and Supporting Information). Thus it is well conceivable that—apart from the potential protection of beetle offspring—gladiofungin or related compounds might play additional ecological roles in other biological contexts. Moreover, it appears that this unusual, thus far overlooked, off‐loading mechanism is employed by other PKS assembly lines. Using the amino acid sequence of the AfsA domain as a bioinformatic handle, we identified various orphan gene clusters coding for (multi)modular assembly lines with end‐standing AfsA domains in genomes of taxonomically distinct microorganisms such as *Gloeobacter*, *Paenibacillus* and *Ruminiclostridium* spp. The encoded biosynthetic pathways likely lead to novel butenolide‐ or γ‐butyrolactone‐substituted natural products (Figure 4 G).

In summary, we succeeded in the genomics‐driven discovery of a potent antifungal natural product (gladiofungin) from a protective beetle symbiont, which may inspire future drug developments. Beyond this pharmacological relevance, this finding is significant from an ecological perspective as it sheds light on the diverse chemical armory of symbiotic bacteria against entomopathogenic fungi that represents the major competitors of the beetle. The novel antifungal polyketide reported herein is highly unusual as it harbors a butenolide moiety that is generated by an unprecedented off‐loading domain. Such a rare component of a multimodular assembly line is an important addition to the biosynthetic toolbox of trans‐AT PKS^[20]^ as it installs structural diversity into complex polyketides. Genome mining suggests that various bacterial taxa have recruited AfsA‐like domains to be used in thiotemplate systems. As it is complementary to currently known avenues,[^18d^, ^21^] the chain‐termination mechanism employed by the *gla* PKS^[22]^ opens new possibilities for synthetic biology approaches. In this context, CRISPR/Cas genome editing of *Burkholderia* was established to enable the precise, marker‐less alteration of a megasynthase.

## Conflict of interest

The authors declare no conflict of interest.

## Supporting information

As a service to our authors and readers, this journal provides supporting information supplied by the authors. Such materials are peer reviewed and may be re‐organized for online delivery, but are not copy‐edited or typeset. Technical support issues arising from supporting information (other than missing files) should be addressed to the authors.

Supplementary

## References

[1a] J. Piel , Nat. Prod. Rep. 2004, 21, 519–538; 15282634 10.1039/b310175b

[1b] J. Piel , Curr. Med. Chem. 2006, 13, 39–50; 16457638

[1c] J. M. Crawford , J. Clardy , Chem. Commun. 2011, 47, 7559–7566. 10.1039/c1cc11574j PMC3174269 21594283

[2a] C. Gimenez , R. Cabrera , M. Reina , A. Gonzalez-Coloma , Curr. Org. Chem. 2007, 11, 707–720;

[2b] S. Kusari , C. Hertweck , M. Spiteller , Chem. Biol. 2012, 19, 792–798; 22840767 10.1016/j.chembiol.2012.06.004

[2c] E. Martinez-Klimova , K. Rodríguez-Peña , S. Sánchez , Biochem. Pharmacol. 2017, 134, 1–17. 27984002 10.1016/j.bcp.2016.10.010

[3a] M. G. Haygood , E. W. Schmidt , S. K. Davidson , D. J. Faulkner , J. Mol. Microbiol. Biotechnol. 1999, 1, 33–43; 10941782

[3b] U. Hentschel , J. Piel , S. M. Degnan , M. W. Taylor , Nat. Rev. Microbiol. 2012, 10, 641–654; 22842661 10.1038/nrmicro2839

[3c] M. Morita , E. W. Schmidt , Nat. Prod. Rep. 2018, 35, 357–378. 29441375 10.1039/c7np00053g PMC6025756

[4a] Y. M. Shi , H. B. Bode , Nat. Prod. Rep. 2018, 35, 309–335; 29359226 10.1039/c7np00054e

[4b] T. R. Ramadhar , C. Beemelmanns , C. R. Currie , J. Clardy , J. Antibiot. 2014, 67, 53–58; 10.1038/ja.2013.77 PMC7381487 23921819

[4c] V. L. Challinor , H. B. Bode , Ann. N. Y. Acad. Sci. 2015, 1354, 82–97. 26509922 10.1111/nyas.12954

[5a] L. V. Flórez , M. Kaltenpoth , Environ. Microbiol. 2017, 19, 3674–3688; 28752961 10.1111/1462-2920.13868

[5b] L. V. Flórez , K. Scherlach , P. Gaube , C. Ross , E. Sitte , C. Hermes , A. Rodrigues , C. Hertweck , M. Kaltenpoth , Nat. Commun. 2017, 8, 15172; 28452358 10.1038/ncomms15172 PMC5414355

[5c] L. V. Flórez , K. Scherlach , I. J. Miller , A. Rodrigues , J. C. Kwan , C. Hertweck , M. Kaltenpoth , Nat. Commun. 2018, 9, 2478. 29946103 10.1038/s41467-018-04955-6 PMC6018673

[6] B. Dose , S. P. Niehs , K. Scherlach , L. V. Flórez , M. Kaltenpoth , C. Hertweck , ACS Chem. Biol. 2018, 13, 2414–2420. 30160099 10.1021/acschembio.8b00600

[7] S. C. Waterworth , L. V. Flórez , E. R. Rees , C. Hertweck , M. Kaltenpoth , J. C. Kwan , mBio 2020, 11, e02430-19. 32098813 10.1128/mBio.02430-19 PMC7042692

[8] J.-y. Kato , N. Funa , H. Watanabe , Y. Ohnishi , S. Horinouchi , Proc. Natl. Acad. Sci. USA 2007, 104, 2378–2383. 17277085 10.1073/pnas.0607472104 PMC1892969

[9] J. A. Gerlt , J. T. Bouvier , D. B. Davidson , H. J. Imker , B. Sadkhin , D. R. Slater , K. L. Whalen , Biochim. Biophys. Acta Proteins Proteomics 2015, 1854, 1019–1037. 10.1016/j.bbapap.2015.04.015 PMC4457552 25900361

[10a] J. Ju , J. W. Seo , Y. Her , S. K. Lim , B. Shen , Org. Lett. 2007, 9, 5183–5186; 17997563 10.1021/ol702249g

[10b] S. K. Lim , J. Ju , E. Zazopoulos , H. Jiang , J. W. Seo , Y. Chen , Z. Feng , S. R. Rajski , C. M. Farnet , B. Shen , J. Biol. Chem. 2009, 284, 29746–29756; 19726666 10.1074/jbc.M109.046805 PMC2785606

[10c] M. Yin , Y. Yan , J. R. Lohman , S.-X. Huang , M. Ma , G.-R. Zhao , L.-H. Xu , W. Xiang , B. Shen , Org. Lett. 2014, 16, 3072–3075. 24815182 10.1021/ol501179w PMC4051428

[11a] B. Kusebauch , B. Busch , K. Scherlach , M. Roth , C. Hertweck , Angew. Chem. Int. Ed. 2010, 49, 1460–1464; 10.1002/anie.200905467 20033973

[11b] S. Sundaram , D. Heine , C. Hertweck , Nat. Chem. Biol. 2015, 11, 949; 26479442 10.1038/nchembio.1932

[11c] T. Bretschneider , J. B. Heim , D. Heine , R. Winkler , B. Busch , B. Kusebauch , T. Stehle , G. Zocher , C. Hertweck , Nature 2013, 502, 124–128. 24048471 10.1038/nature12588

[12a] S. R. Rajski , B. Shen , ChemBioChem 2010, 11, 1951–1954; 20806307 10.1002/cbic.201000370 PMC3517116

[12b] N. Saito , F. Suzuki , K. Sasaki , N. Ishida , Antimicrob. Agents Chemother. 1976, 10, 14–19. 984746 10.1128/aac.10.1.14 PMC429681

[13a] B. Wang , Y. Song , M. Luo , Q. Chen , J. Ma , H. Huang , J. Ju , Org. Lett. 2013, 15, 1278–1281; 23438151 10.1021/ol400224n

[13b] D. Heine , T. Bretschneider , S. Sundaram , C. Hertweck , Angew. Chem. Int. Ed. 2014, 53, 11645–11649; 10.1002/anie.201407282 25214315

[14] J.-W. Seo , M. Ma , T. Kwong , J. Ju , S.-K. Lim , H. Jiang , J. R. Lohman , C. Yang , J. Cleveland , E. Zazopoulos , Biochemistry 2014, 53, 7854–7865. 25405956 10.1021/bi501396v PMC4270375

[15] P. Caffrey , ChemBioChem 2003, 4, 654–657. 12851937 10.1002/cbic.200300581

[16] A. Kitsche , M. Kalesse , ChemBioChem 2013, 14, 851–861. 23576424 10.1002/cbic.201300063

[17] K. Arakawa , N. Tsuda , A. Taniguchi , H. Kinashi , ChemBioChem 2012, 13, 1447–1457. 22761035 10.1002/cbic.201200149

[18a] M. Klapper , K. Schlabach , A. Paschold , S. Zhang , S. Chowdhury , K.-D. Menzel , M. A. Rosenbaum , P. Stallforth , Angew. Chem. Int. Ed. 2020, 59, 5607–5610; 10.1002/anie.201914154 PMC7154651 31880848

[18b] Z. Salehi-Najafabadi , C. Barreiro , A. Rodríguez-García , A. Cruz , G. E. López , J. F. Martín , Appl. Microbiol. Biotechnol. 2014, 98, 4919–4936; 24562179 10.1007/s00253-014-5595-9

[18c] J. D. Sidda , C. Corre , Methods Enzymol. 2012, 517, 71–87; 23084934 10.1016/B978-0-12-404634-4.00004-8

[18d] Y. Demydchuk , Y. Sun , H. Hong , J. Staunton , J. B. Spencer , P. F. Leadlay , ChemBioChem 2008, 9, 1136–1145. 18404760 10.1002/cbic.200700715

[19] X. Wang , H. Zhou , H. Chen , X. Jing , W. Zheng , R. Li , T. Sun , J. Liu , J. Fu , L. Huo , Proc. Natl. Acad. Sci. USA 2018, 115, E4255–E4263. 29666226 10.1073/pnas.1720941115 PMC5939090

[20] E. J. N. Helfrich , J. Piel , Nat. Prod. Rep. 2016, 33, 231–316. 26689670 10.1039/c5np00125k

[21a] Y. Sun , F. Hahn , Y. Demydchuk , J. Chettle , M. Tosin , H. Osada , P. F. Leadlay , Nat. Chem. Biol. 2010, 6, 99; 20081823 10.1038/nchembio.285 PMC2811812

[21b] L. Vieweg , S. Reichau , R. Schobert , P. F. Leadlay , R. D. Süssmuth , Nat. Prod. Rep. 2014, 31, 1554–1584. 24965099 10.1039/c4np00015c

[22] See also the concurrent, independent study: I. T. Nakou , M. Jenner , Y. Dashti , I. Romero-Canelón , J. Masschelein , E. Mahenthiralingam , G. Challis , Angew. Chem. Int. Ed. 2020, 10.1002/anie.202009007; PMC7756379 32918852

