# Supplementary material for: Insect‐Associated Bacteria Assemble the Antifungal Butenolide Gladiofungin by Non‐Canonical Polyketide Chain Termination
Source: Angew Chem Int Ed Engl. 2020 Oct 15;59(51):23122–6. doi: 10.1002/anie.202005711 (PMC7756420; doi:10.1002/anie.202005711)
Supplement: Supplementary file 1 — Supplementary [file ANIE-59-23122-s001.pdf]

Angewandte Chemie
Eine Zeitschrift der Gesellschaft Deutscher Chemiker

Supporting Information

Insect-Associated Bacteria Assemble the Antifungal Butenolide Gladiofungin by Non-Canonical Polyketide Chain Termination

*Sarah P. Niehs[+], Jana Kumpfmüller[+], Benjamin Dose, Rory F. Little, Keishi Ishida, Laura V. Flórez, Martin Kaltenpoth, and Christian Hertweck**

anie_202005711_sm_miscellaneous_information.pdf

Table of Contents

Strains, culture conditions, and extraction of metabolites 3
Annotation of the gladiofungin biosynthesis gene cluster 3
Gene cluster comparison using EasyFig2.3 5
Sequence similarity network (SSN) and genome neighborhood network (GNN) 5
Knockout-mutant generation by *kanR* insertion into *glaD*, and *glaG* 8
Generation of CRISPR/Cas mutants containing a mutated or deleted AfsA domain 9
Transcriptome analysis of the *B. gladioli afsA*-del mutant 17
Phylogenetic analysis of ketosynthase domains of gladiofungin PKS 19
Determining the ketoreductase specificities 20
General analytical methods 20
Isolation of gladiofungins 20
Labeling experiments 21
Determination of bioactivity 21
MS/MS spectra 23
NMR tables 24
Detailed structure elucidation 26
NMR spectra 27
Supplemental References 34

Supplemental Tables

Table S1. Composition of media used in this study. 3
Table S2. Deduced gene functions. 4
Table S3. Strains used in this study. 11
Table S4. Plasmids used in this study. 11
Table S5. Oligonucleotides used for the construction and verification of gladiofungin mutants 12
Table S6. Sequences of cassettes for genome editing by CRISPR/Cas. 13
Table S7. Sequences of the target and the most likely off-targets for the *afsA*-guide RNA. 16
Table S8. Inhibitory effects of gladiofungin A against several bacterial and fungal strains. 21
Table S9. Agar diffusion assay against *Purpureocillium lilacinum* with inhibition zone. 22
Table S10. Cytotoxic and antiproliferative profiling of gladiofungin A. 22
Table S11. NMR shifts of gladiofungin A (1). 24
Table S12. ^{1}H NMR shifts of gladiofungin B (2). 25

Supplemental Figures

Figure S1. Biosynthetic origin of gladiofungins and other glutarimides.. 6
Figure S2. Comparison of glutarimide assembly lines in *Burkholderia* spp. and *Streptomyces* spp. 7
Figure S3. Conservation of gladiofungin-like assembly lines. 7
Figure S4. Construction of gene deletion mutant Δ*glaD*. 9
Figure S5. Maps of plasmids used for genome editing. 14
Figure S6. DNA sequence chromatogram verifying the precise deletion of the *afsA* domain region in *B. gladioli afsA*-del. 15
Figure S7. Comparison of DNA sequence chromatograms verifying the precise mutation of the *afsA* domain region in two obtained *B. gladioli afsA*-PM mutants. 15
Figure S8. DNA sequence chromatograms verifying the unaltered gene sequence of the most-likely off-target regions. 16
Figure S9. Metabolic profiles of *B. gladioli* HKI0739 wild type and *afsA*-PM as total ion chromatogram (TIC) and extracted ion chromatogram (EIC). 17
Figure S10. Growth curve of the *B. gladioli* WT and *B. gladioli afsA*-del strain for mRNA analysis. 18
Figure S11. Qualitative RT-PCR-based verification of the expression of *glaF* and *glaG* (A), *glaD* (B), and the *afsA*-region (C) in the *B. gladioli* WT and *B. gladioli afsA*-del strain. 18
Figure S12. Maximum Likelihood tree for ketosynthase specificity....................................... 19
Figure S13. Agar diffusion assay for *Purpureocillium lilacinum*. 22
Figure S14. MS/MS fragmentation pattern of gladiofungin A (**1**) (*m/z* 504.2604 [M–H]$^-$). 23
Figure S15. MS/MS fragmentation pattern of gladiofungin B (**2**) (*m/z* 502.2448 [M–H]$^-$). 23
Figure S16. ^{1}H NMR spectrum of gladiofungin A (**1**). 27
Figure S17. ^{13}C NMR spectrum of gladiofungin A (**1**). 28
Figure S18. DEPT135 NMR spectrum of gladiofungin A (**1**)... 28
Figure S19. ^{1}H-^{1}H COSY NMR spectrum of gladiofungin A (**1**). 29
Figure S20. ^{1}H-^{13}C HMBC NMR spectrum of gladiofungin A (**1**). 30
Figure S21. ^{1}H-^{13}C HSQC NMR spectrum of gladiofungin A (**1**).. 31
Figure S22. ^{1}H NMR spectrum of gladiofungin B (**2**). 32
Figure S23. ADEQUATE NMR spectrum of gladiofungin A (**1**)... 32
Figure S24. INADEQUATE NMR spectrum of ^{13}C-labeled gladiofungin A (**1**). 33

Supplemental References

Strains, culture conditions, and extraction of metabolites

Burkholderia gladioli HKI0739 was cultivated on PDA. The bacteria were stored in 50% glycerol at –20 °C. Culture media recipes are presented in Table S1. Prior to use all media were sterilized at 121 °C for 20 min.

For large-scale purification of gladiofungins, *B. gladioli* HKI0739 was plated onto NAG agar and grown at 30 °C. Colonies were transferred to MGY+M9 medium and grown to late exponential phase at 30 °C and with shaking at 110 rpm. This bacterial culture was inoculated in 400 mL PDB medium in 1-L-Erlenmeyer-flasks (14 flasks; 5.6 L total) until an OD_{600} 0.05 was reached. Subsequently, the culture was grown for 24 h at 30 °C, 120 rpm. The bacterial cultures were then extracted using an equal volume of ethyl acetate overnight. The organic phase was dried over sodium sulfate and concentrated to dryness under reduced pressure. The residue was dissolved in methanol. LC/HR-MS analysis of the extracts revealed *m/z* 506.2756 $[M+H]^+$ for gladiofungin A (calculated for 506.2748, $C_{27}H_{40}NO_8$) and *m/z* 504.2606 $[M+H]^+$ for gladiofungin B (calculated for 504.2592, $C_{27}H_{38}NO_8$).

Table S1. Composition of media used in this study.

**Medium or component**	**Composition (L^{-1})**
LB medium/agar	10 g Tryptone (BD, Bacto), 5 g yeast extract (technical yeast extract, BD, Bacto®), 10 g NaCl, sterilization; for agar: addition of 1.5% agar (w/v)
MGY+M9 medium	10 g Glycerol, 1.25 g yeast extract (technical yeast extract, BD, Bacto®), 960 mL water, sterilization, then add: 20 mL M9 salt A, 20 mL M9 salt B
NAG agar	Standard Nutrient I agar (BD, Bacto®), 1% glycerol (w/v), sterilization
PDA	Potato dextrose agar (BD, Bacto®), sterilization
PDB	Potato dextrose broth (BD, Bacto®), sterilization
M9 salt solution A	350 g K_2HPO_4, 100 g KH_2PO_4
M9 salt solution B	29.4 g Sodium citrate, 50 g $(NH_4)_2SO_4$, 5 g $MgSO_4$

Annotation of the gladiofungin biosynthesis gene cluster

AntiSMASH version 5.0[1] and PKS/NRPS analysis[2] were used to analyze the genome sequence of *B. gladioli* HKI0739 (Figure 1A). NCBI BLAST (database: swissprot) or HHpred[3] (databases: PDB_mmCIF70_27_Apr, Pfam-A_v32.0, NCBI_Conserved_Domains(CD)_v3.18) were used to identify unusual domains and closest homologues of described proteins (Table S2).

Table S2. Deduced gene functions.

gla	Length [bp]	Putative function	Closest characterized orthologous protein (HHpred or swissprot BLAST*) [Species]	Accession number	Identity/ similarity
T	1,263	Transporter	Bacterial MdtG-like and eukaryotic solute carrier 18 (SLC18) family of the Major Facilitator Superfamily of transporters [-]	cd17325	17%/13%
K	720	Hypothetical protein	UPF0502 protein Bmul_3231/BMULJ_05293 [*Burkholderia multivorans*]	5VYV_A	43%/64%
P	702	Phosphatase	Glucosyl-3-phosphoglycerate phosphatase [*Mycobacterium tuberculosis*]	4PZA_B	23%/29%
A	3,360	Fatty acid synthase	Polyketide biosynthesis protein BaeE [*Bacillus velezensis*]*	A7Z4Y0	53%/69%
B	243	Acyl carrier protein	D-Alanyl carrier protein [*Streptococcus sanguinis*]*	A3CR85	36%/51%
C	1,998	Amidotransferase	Asparagine synthetase [*Homo sapiens*]	6GQ3_A	25%/43%
D	9,747	Polyketide synthase	Polyketide synthase PksL [*Bacillus subtilis* subsp. *subtilis*]*	Q05470	38%/54%
E	30,546	Polyketide synthase	Polyketide synthase PksN [*Bacillus subtilis* subsp. *subtilis*]*,[a]	O31782	41%/58%
F	714	Hypothetical protein	Hydrolase, haloacid dehalogenase-like family [*Pseudomonas syringae* pv. *tomato*]	3S6J_F	13%/7%
G	1,023	Enoylreductase	Enoylreductase [*Mycobacterium smegmatis*]	5BP4_R	22%/24%
R	909	Regulatory protein	Hydrogen peroxide-inducible genes activator; transcription factor [*Corynebacterium glutamicum*]	6G1D_B	15%/24%
H	873	Dehydrogenase	3-hydroxyisobutyrate dehydrogenase [*Salmonella typhimurium*]	3G0O_A	27%/43%
I	1,518	Dehydrogenase	D-glyceraldehyde dehydrogenase [*Thermoplasma acidophilum*]	5IZD_E	27%/49%
J	1,335	Hypothetical protein	Protein of unknown function (DUF3100) [-]	PF11299.8	41%/73%

[a] HHpred: Module 10 with domains showing similarities to a) Polyketide synthase PksJ [*Bacillus subtilis* subsp. *subtilis*], 4NA1_B, 56%/99%, b) Tyrocidine synthetase 3 [*Brevibacillus brevis*], 2JGP_A, 14%/11%, and c) AfsA ; A-factor biosynthesis hotdog domain [-], PF03756.13, 13%/9%

Gene cluster comparison using EasyFig2.3

The sequence similarity of gene clusters encoding glutarimide-forming biosynthetic assembly lines is visualized using the tool Easyfig 2.3. Color code representing the sequence similarity values is given in the figure. GC base content is shown for the gladiofungin biosynthesis gene cluster (Figure S1A–B).

Sequence similarity network (SSN) and genome neighborhood network (GNN)

Sequence similarity networks and genome neighborhood networks were created using the enzyme function initiative: enzyme similarity tool (EFI-EST) and genome neighborhood tool (EFI-GNT).[4] The amino acid sequences of GlaE or GlaD (module 1 and 2) were submitted as the queries to the EFI-EST online tool to create SSNs. Subsequently, the results were forwarded to EFI-GNT to create the corresponding the genome neighborhood. The SSNs were manually inspected for clusters in which glutarimide biosynthesis machineries are encoded (Figure S1B).

Detailed analysis of *gla* PKS:

For a detailed analysis of the *gla* PKS, the deduced amino acid sequences of GlaABDE were submitted as query to the EFI-EST (enzyme function initiative: enzyme similarity tool)[4] to create SSNs (sequence similarity networks), and the results were forwarded to the genome neighborhood tool (EFI-GNT). The SSN generated with GlaE (AfsA domain) did not reveal any correlations to other biosynthetic gene clusters than those of *B. gladioli*. The SSN using the amino acid sequence of GlaD as query, however, revealed three significant neural clouds (Figure S1B): 1) three homologous gene clusters from related *Burkholderia* strains; 2) a biosynthetic gene cluster (BGC) in *Streptomyces amphibiosporus* for lactimidomycin (**6**)[5] one in *Streptomyces platensis* for iso-migrastatin (**7**) assembly,[5] and 14 similar gene clusters in *Streptomyces* spp. or related strains; 3) the streptimidone (**8a, 8b**) BGC in *Streptomyces himastatinicus*,[6] the BGC for cycloheximide (**9**) and actiphenol (**10**) in *Streptomyces* sp. YIM 56141,[7] and 42 similar gene clusters in other *Streptomyces* spp. or related strains (Figure S1A–C). Notably, all of the known encoded compounds (**6–10**) share a glutarimide pharmacophore.

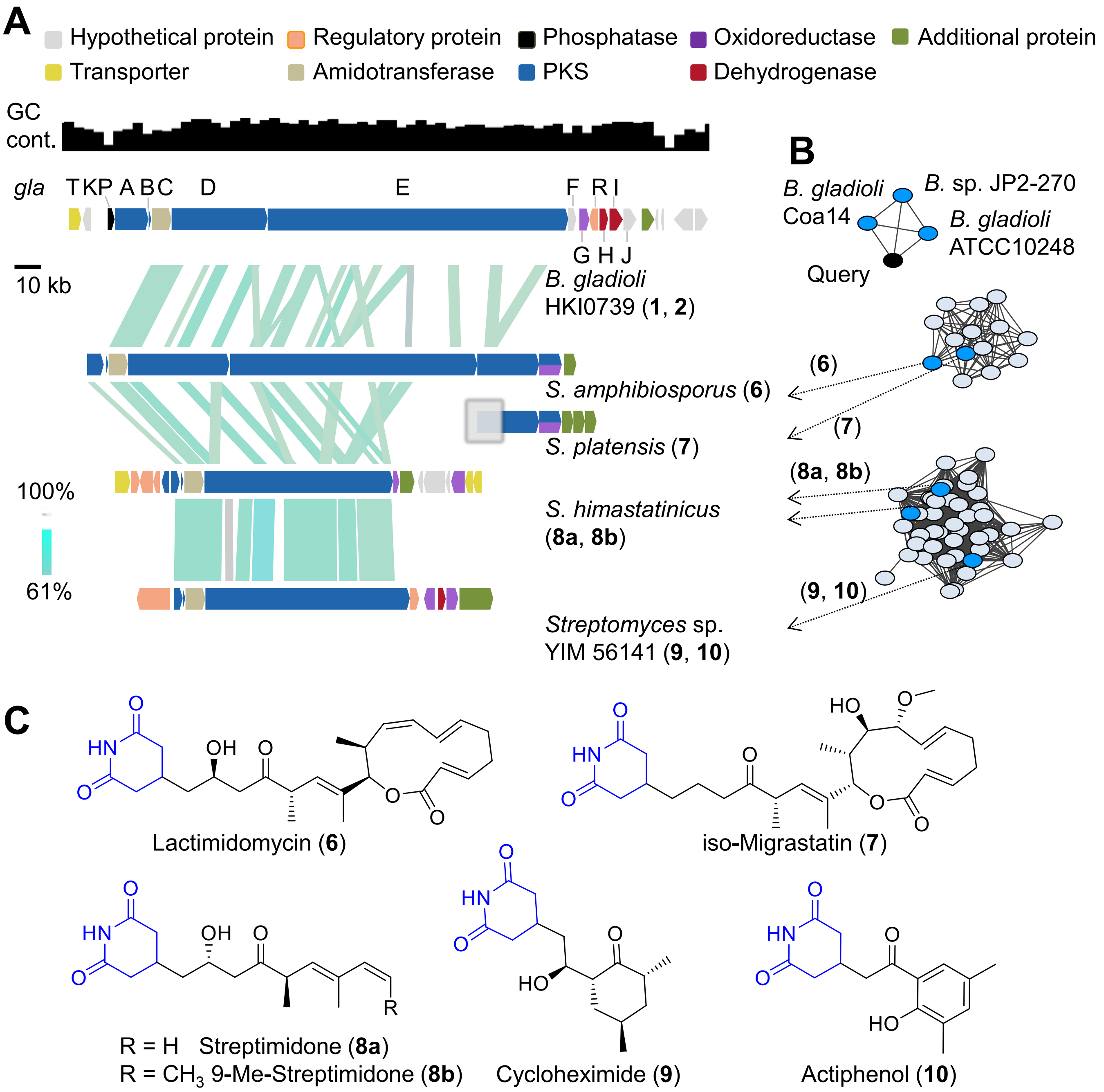

Figure S1. Biosynthetic origin of gladiofungins and other glutarimides. A) Cryptic *B. gladioli* BGC with similarities to *Streptomyces* BGCs. BGC boundaries according to GC content drops. B) Sequence similarity network created with the deduced amino acid sequence of GlaD as queries to the EFI-EST web tool. Dark blue, known gene cluster. Black, query. Light blue, orphan gene cluster. C) Selection of natural products containing a glutarimide moiety.

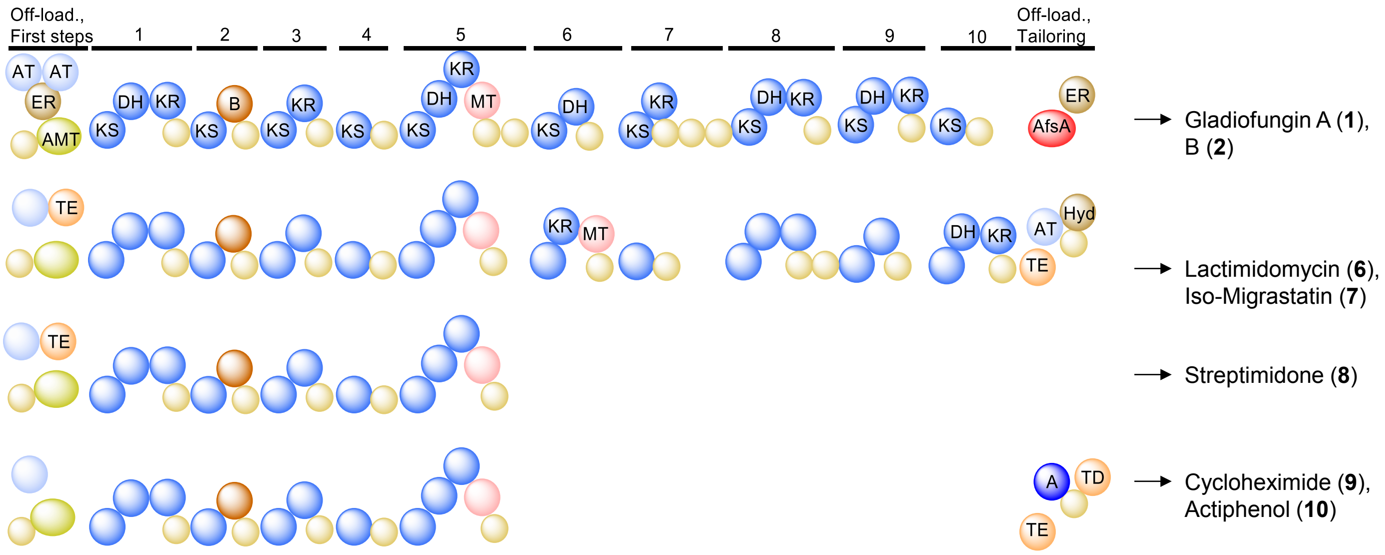

Figure S2. Comparison of glutarimide assembly lines in *Burkholderia* spp. and *Streptomyces* spp. KS, ketosynthase; KR, ketoreductase; DH, dehydratase; ER, enoyl reductase; AT, acyl transferase; B, β-branching domain; TE, thioesterase; A, adenylation domain; Hyd, hydrolase; yellow, ACP, acyl carrier protein; AMT, amidotransferase.

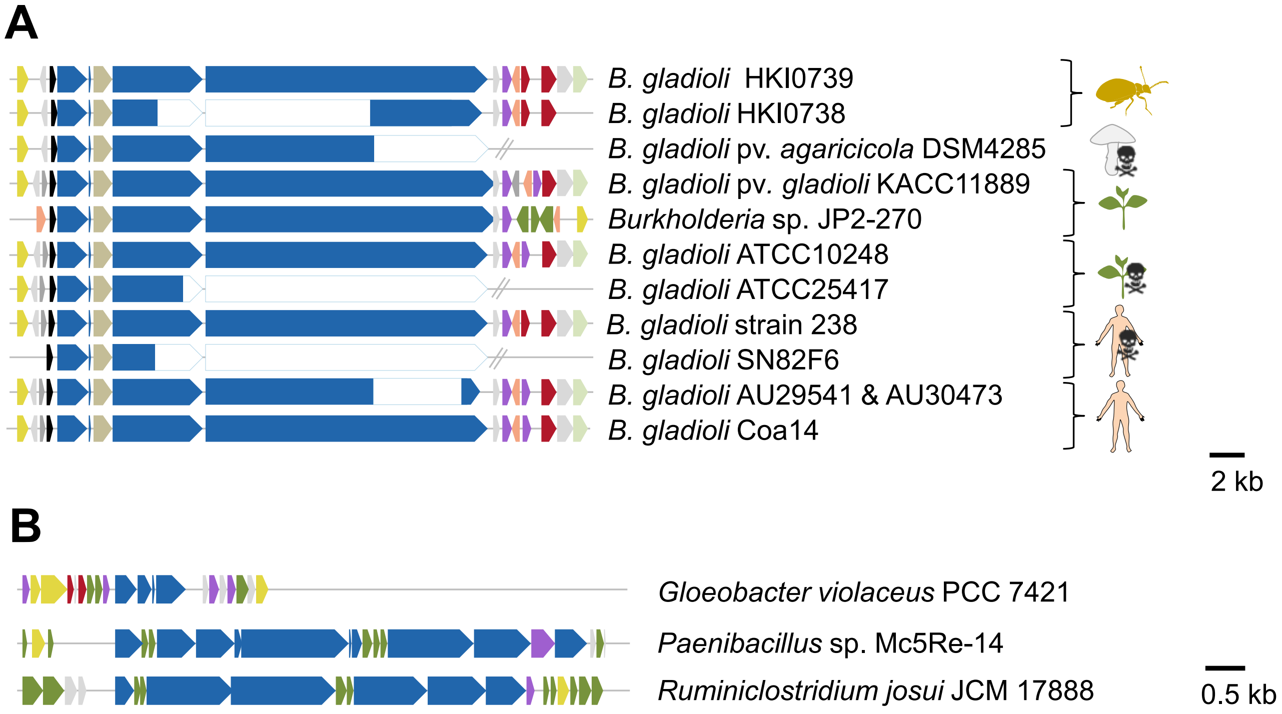

Figure S3. Conservation of gladiofungin-like assembly lines. A) Conserved gladiofungin BGC in *Burkholderia* strains from diverse niches. White arrows indicate poor genomic sequence. B) Additional PKS assembly lines with an endstanding AfsA domain. Color code according to Figure 1; gray, hypothetical gene; yellow, genes coding for transporter; orange, regulators, light brown, amidotransferase; black, phosphatase; blue, PKS; purple, oxidoreductase; dark red, dehydrogenase; green, additional genes.

Knockout-mutant generation by *kanR* insertion into *glaD*, and *glaG*

Plasmids used in this section (see Table S4 and Figure S4) were designed to chromosomally integrate a kanamycin resistance marker (*kanR*) cassette into *glaD* (pJK345), or *glaG* (pJK347) via homologous recombination. Homologous flanking regions (F1 and F2) of the gene of interest were amplified by Phusion polymerase (New England Biolabs) from *B. gladioli* HKI0739 genomic DNA using the primers listed in Table S5. All flanking regions amplified were between 500 to 650 bp in size. The *kanR* cassettes were amplified from pGEM-Kan[8] by OneTaq polymerase (New England Biolabs) using the primers listed in Table S5. For the *glaD* and *glaG* knockout plasmids pJK345 and pJK347, *Xba*I/*Xho*I-digested pJET1.2 (Thermo Fisher Scientific) was assembled with the specific flanking regions F1 and F2 as well as the corresponding *kanR* cassette in a one-step reaction using NEBuilder HiFi DNA Assembly mix (New England Biolabs).

Following creation of the gene deletion plasmids, fresh *B. gladioli* HKI0739 cells growing on NAG agar were inoculated into 20 mL of MGY+M9 medium and incubated at 30 °C and with shaking at 110 rpm until cells reached OD_{600} of 0.8–1.4. The cells were harvested by centrifugation at 6,000 × g at room temperature for 5 min and the precipitated cells were washed twice in 20 mL MiliQ water. The resulting cell pellet was resuspended in 500 µL MiliQ water. For each transformation, 100 µL of these cells were transformed with 0.5–1 µg of pJK345, pJK347, or pJK348 by electroporation. All electroporations were performed at 2.5 kV in a 2 mm gapped electroporation cuvette, followed by the addition of 500 µL of MGY+M9 medium. After incubation at 30 °C and with shaking at 100 rpm for 3 h, the cells were plated on NAG-kanamycin (300 µg mL^{-1}) agar plate and incubated at 30 °C for 2 days.

Chromosomal integration of the kanamycin marker gene into *glaD/G* was verified by colony PCR using KAPA2G Robust HotStart ReadyMix PCR kit (Sigma-Aldrich). The primers were designed to detect the integration of the *kanR* cassette into Δ*glaD* (JK556/Kan-seq_rv), or Δ*glaG* (JK573/Kan-seq_rv), respectively. All positive colonies were counter screened to ensure they lacked any wild-type contamination and were not merely single crossover mutants (using the primers JK556/JK575 for pJK345, and JK560/JK574 for pJK347). Finally, positive colonies of each mutant were selected and the entire mutated gene region amplified for sequencing (Genewiz) and further analysis (using primer pairs JK556/JK557 for Δ*glaD*, and JK560/JK561 for Δ*glaG*). The obtained mutants are listed in Table S3. A scheme as well as the products of the final PCR for generation of the *glaD* mutant is shown in Figure S4.

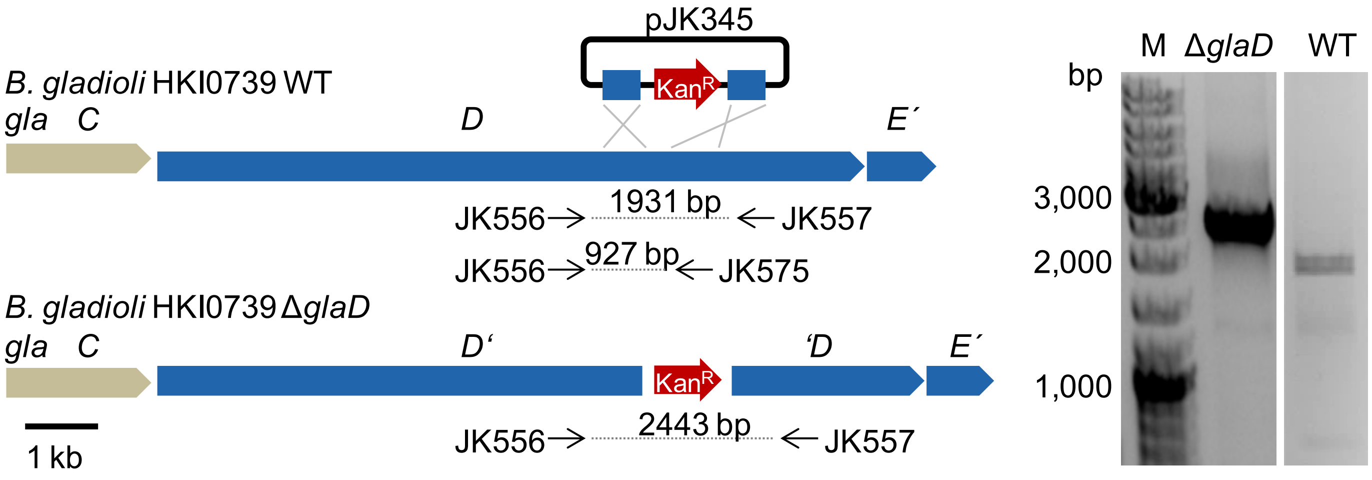

Figure S4. Construction of gene deletion mutant Δ*glaD*. M, marker. Kan^R, kanamycin-resistance cassette. WT, wild type.

Generation of CRISPR/Cas mutants containing a mutated or deleted AfsA domain

To establish a working CRISPR/Cas system for *Burkholderia gladioli*, two methods were combined: 1) A rhamnose inducible *Burkholderia*-optimized red-operon to enhance homologous recombination;[9] and 2) A single-copy, inducible, codon-optimized *cas9*[10] to cause a double-strand break at a specific position as determined by the N20-sequence of the sgRNA. All strains, plasmids, and primers used in this section are listed in Tables S3–5, respectively. The temperature-sensitive low-copy plasmid with a *Burkholderia*-optimized recombination system, pTsC-Red, was derived as follows. First, the temperature-sensitive pRO1600(Ts) replicon[11] and the genes coding for RedBA7029[9] were constructed by gene synthesis (Life Technologies, ThermoFisher). Plasmid pMK-TsOriRO1600 was then *Bsp*HI/*Fsp*I-digested and ligated with a *Bsp*HI-digested chloramphenicol resistance marker (*cmR*) cassette amplified from pCR-*cmR* (a subcloning plasmid that contains the *cmR* cassette from pACYCDuet-1 from Novagen) using the primers JK435/JK436 to yield plasmid pMC-TsOriRO1600. Next, *redγ* was amplified from pKD46-Gm[12] using the primers JK397/JK398 and *redβα7029* was amplified from pMK-RedBA7029 using the primers JK399/JK400. Both PCR products were assembled into *Nde*I/*Sbf*I-digested pSCrhaB2 to give pRedBA7029. Finally, the *rhaSR*-P_{rhaB}-red*γβα7029* cassette was cut from pRedBA7029 using *Bsr*GI/*Sbf*I and ligated in between the *Bsr*GI/*Sbf*I sites of pMC-TsOriRO1600 to create pTsC-Red, a helper plasmid that can be used for homologous recombination in diverse *Burkholderia* strains.

To create pTsK-CasRed-Bt, a one-step CRISPR/Cas plasmid based on pTsC-Red, a codon-optimized, *Streptococcus pyogenes* derived *cas9** gene in combination with the *rhaB* promoter was synthesized (Genewiz). The codon-optimization analysis was performed by the GENEius web tool (http://www.geneius.de) using the codon-usage table of *Burkholderia thailandensis*. The *cmR* cassette from pTsC-Red was replaced by a *kanR* cassette by digesting pTsC-Red using *Psp*OMI/*Bsp*EI and inserting the *kanR* cassette (amplified from pGEM-Kan[8] using the primers JK678/679) using NEBuilder isothermic assembly, producing pTsK-Red. Next, the P_{rhaB}-*cas9**-cassette was cut from its subcloning vector by digestion with *Psp*OMI/*Sbf*I and ligated into pTsK-Red, cut with the same restriction enzymes, to yield pTsK-CasRed-Bt.

Cassettes containing the specific guide RNA (sgRNA) for attack at the N20-PAM sequence CAT CGA CTG ACG GGC AGC CT-CGG as well as the alternative DNA for genome editing were obtained by gene synthesis (Genewiz) and are displayed in Table S6. These cassettes were excised from their subcloning plasmids using *Psp*OMI/*Xho*I and ligated into pTsK-CasRed-Bt, yielding plasmids pJK363 for *afsA* point mutation (PM) and pJK364 for *afsA*-domain deletion, respectively (Figure S5). Both plasmids were introduced into *B. gladioli* HKI0739 cells by electroporation as described above. Cells were plated on NAG-kanamycin (300 µg mL^{-1}) agar supplemented with 2.5 mg mL^{-1} L-rhamnose (NAG-Kan-Rha) and incubated at 30 °C for 2 days. Up to 30 kanamycin-resistant colonies were picked on a fresh NAG-Kan-Rha plate and again incubated at 30 °C for 2 days. This was repeated twice until colonies displaying reduced growth were detected. These colonies were checked by colony-PCR using primers JK725/JK610 (protocol A). The *afsA* domain coding-frame deletion mutant was detected by the expected product size (1,401 bp instead of 1,971 bp for the wild type). To identify the *afsA*-PM mutant (E10080A), the PCR product was digested with *Sac*II. Clones containing the point mutation showed a fragment pattern of 837 bp + 354 bp + 406 bp + 374 bp instead of the wild-type pattern of 1,191 bp + 406 bp + 374 bp. To verify that wild-type contamination was absent, all positive colonies were streaked out on NAG-Kan-Rha plate again, and single colonies were checked using the same method as before. In addition, a second PCR using primers JK725/JK728 (protocol B) was performed that only gave a product (856 bp) if contaminating wild type cells were present. To cure the plasmid, 1–2 positive colonies were streaked on a NAG agar plate (without kanamycin and rhamnose) and incubated at 37 °C for 16 hours. Grown colonies were tested for kanamycin sensitivity, indicating plasmid loss. Again, positive colonies were screened using colony PCR (protocols A and B) to ensure the correct genotype and the absence of wild type cells. For final verification, the product of PCR protocol A was sequenced (Genewiz) (Figure S6 and Figure S7).

Table S3. Strains used in this study.

Strains	Description	Source
Burkholderia gladioli HKI0739	Wild-type, environmental isolate	[13]
B. gladioli Δ*glaD*	*glaD*:: KanR	This study
B. gladioli Δ*glaG*	*glaG*:: KanR	This study
B. gladioli afsA-PM	*glaE* E10080A	This study
B. gladioli afsA-del	*glaE* Δ*afsA*	This study

KanR – kanamycin resistance

Table S4. Plasmids used in this study.

Name	Description	Marker	Size	Reference
**KanR insertion plasmids**				
pJK345	for KanR insertion into *glaD*	AmpR, KanR	5.2 kb	This study
pJK347	for KanR insertion into *glaG*	AmpR, KanR	5.2 kb	This study
**λ-Red and CRISPR/Cas plasmids**				
pMC-TsOriRO1600	Temperature-sensitive shuttle vector for *Burkholderia* (low copy)	CmR	3.3 kb	This study
pSCrhaB2	Shuttle vector for *Burkholderia* containing genetic elements for L-rhamnose inducible gene expression, pBBR1 replicon	TmR	7.5 kb	[14]
pRedBA7029	pSCrhaB2 with a *Burkholderia*-optimized Red-operon for homologous recombination (Redγβα7029)	TmR	9.4 kb	This study
pTsC-Red	pMC-TsOriRO1600 with genetic elements for L-rhamnose inducible expression of Redγβα7029	CmR	7.3 kb	This study
pTsK-Red	pTsC-Red with KanR instead of CmR	KanR	7.8 kb	This study
pTsK-CasRed-Bt	pTsK with codon-optimized *cas9** under control of the *rhaB* promoter	KanR	12.1 kb	This study
pJK363	1-Step CRISPR/Cas vector for *afsA* point mutation (E10080A)	KanR	13.3 kb	this study
pJK364	1-Step CRISPR/Cas vector for *afsA* deletion	KanR	13.3 kb	this study

AmpR – Ampicillin resistance; KanR – kanamycin resistance; CmR – chloramphenicol resistance; TmR – trimethoprim resistance

Table S5. Oligonucleotides used for the construction and verification of gladiofungin mutants.

Name	Sequence 5' → 3' (primer binding site)	Purpose	
**Plasmid construction**			
JK534	GCCAGATCTTCCGGATGGCTCGAGCTGGCCGAGTGGCGGC	F1 for *glaD*	for pJK345
JK535	CCCTCGGCCCAATGACGCGC		
JK536	GCGCGTCATTGGGCCGAGGGCGCGCGGCTCGACTGGCGCCTGCTGTGGCCCGAGCGGGCGCCGCATCGCCTGTCGCTGCCGCCAGTCCGCAGAAACGGTG	KanR for *glaD*	
JK537	GCGAGGAAGTGCGCGACCTGCTGCACGGCGCCGAGCACGGCCGGCGCGAGCGCGACGCGCGCGGCGAAGTGGTGTTGCTTCGGCCGCGGGAATTCGATTC		
JK538	CAGGTCGCGCACTTCCTCGC	F2 for *glaD*	
JK539	GAATATTGTAGGAGATCTTCTAGACCTCGAAGAACACTGCCG		
JK548	GCCAGATCTTCCGGATGGCTCGAGGTGGTGGACTACCTTC	F1 for *glaG*	for pJK347
JK549	CATCGATTTCCTGCAGCAAG		
JK550	CTTGCTGCAGGAAATCGATGCCGCCGCCGTGCATGGAGGCGGCTGGTGAGCCCCGCCCGCGAGCGCCGGGGGAGGGCCACGCCAGTCCGCAGAAACGGTG	KanR for *glaG*	
JK551	GCTTGTGTGTGCCATGTGCAGCCTGTCTCTACTCGTTCCCGCCCTCAAGCGACCAGCTTCACCACCTGCTTGCCATGATTACCGCGGGAATTCGATTCAG		
JK552	TGCACATGGCACACACAAGC	F2 for *glaG*	
JK553	ATTGTAGGAGATCTTCTAGAGGCTCGGCATCTCGGTGATG		
JK397	GAAATTCAGCAGGATCA*CATATG*GATATTAATACTGAAAC	*redγ* amplification	for pRedBA 7029
JK398	GGTCATTTCGTTTTATACCTCTGAATCAATATC		
JK399	CAGAGGTATAAAACGAAATGACCAACGCCCTCACG	*redβα7029* amplification	
JK400	CAAAACAGCC*AAGCTTGCATGCCTGCAGG*TCATGCTGCCTC		
JK435	ATTTTGGTCATGAGACGTTGATCGGCACG	CmR amplification for pMC-TsOriRO1600	
JK436	GCA*CCTGCAGGACTAGCGCTACTCTAGATATCGCGATCGC*G		
JK678	CCTGATGAATGCTCA*TCCGGA*TGAATGTCAGCTACTGG	KanR amplification for pTsK-Red	
JK679	CTCGAGCATGCTGCA*GGGCCC*GCTCAGAAGAACTCGTC		
**Mutant verification and RT-PCR**			
JK556	GAATCCGGCGATCCCGTTCG	check Δ*glaD*	
JK557	CGTGGTTGCTCATCAGATGC		
JK575	GGTCCGCTCCAGTCCGGCTCC		
JK560	GCTGTGCAGCGAGTATTACC	check Δ*glaG*	
JK561	CCTCAAGGAGTTCGTGAAG		
JK574	CATAGCTCTTGGATACGCTC		
Kan-seq_fw	GGCTACCCGTGATATTGC	check KanR insertion mutants	
Kan-seq_rv	GCTTCCCAACCTTACCAGAG		
JK610	GAATCCGGCGATCCCGTTCG	check *afsA* mutants	
JK725	CAAGACGCAGGGCGTGAC		
JK728	CATCGACTGACGGGCAGCCT		
**Off-target verification**			
JK837	CAAGGATCTCGGCAACGTGG	check ctg1 CDS_652	
JK838	GCCCAGCAGGTTGGTCACCG		
JK839	ATGAGCGGCGTGCCGATCGC	check ctg1 CDS_2223	
JK840	GAAGGCACGCATCAGGATGG		
JK841	CGTGAATGCCGCGCTCAACC	check ctg2 CDS_2766	
JK842	GCCAGTGGTCGTGCCCAGCC		

Table S6. Sequences of cassettes for genome editing by CRISPR/Cas.

Target	Sequence of gene synthesis cassettes
afsA<br>→ PM	*GGGCCC*GAGCTCTTGACAGCTAGCTCAGTCCTAGGTATAATGCTAGC**CATCGACTGACGGGCA**<br>**GCCT**GTTTTAGAGCTAGAAATAGCAAGTTAAAATAAGGCTAGTCCGTTATCAACTTGAAAAAGTG<br>GCACCGAGTCGGTGCTTTTTTTGAATTCTCTAGACGAATCGCACGCGTTCTTCTTCGACCATCC<br>GCTCGACCATGTGTCCGGCCTGCATCTGGGCGCGGCGATGAGCGAGGCGGTGCAGGCGGCC<br>CATCTGCATCGGCACGGCCTGCCGGCCGACACCGCGCTGTTCGTATCGCAAGTGCGGCTGCA<br>CTTCGTCGATCTGTGCCGCAAGGAGCCCGGCGCGACGATCCATGTGAGCGCCGCCGCCGGCG<br>CATCCGACACGTATCAGGCGCAGGTGGTCCAGAACGACCGCGAGATGGCTCACGCCGAGTTC<br>GTGGTGAGCGCGGTGCCGCCGCGTCCGCCGCGCGCCGTCGGCGAGATGCCCGGGCGCGCC<br>GAGCCGGCGCCCGCGCGCAGCCTCAACAAGCACGATGCGGCAAACGTGCTGCTCAGCGAGGT<br>GCGCGGCGAGGCCGGCCAGCCGAGCTGCGCGCTGGTGATGCAGCCGGACAACGCGTATTTC<br>GGCGATTTCCCGGCGTCCTGGATCGACATCGTGGTGCTGG*CC*_**GCGG**_**CGGCACGGCAGT**CGAT<br>GCGGCTGTTTACCGCCGAGCGCCGGCTGGCGGCCAGCCTGCCGGCACGCGGCGAGGCCACC<br>CGCGACGTGCTGAAGGCGCTGCACGTCGCGCTGGATCGGCCGCTGTACTGGGACGACGCCGT<br>GGAGCTGGTGTTCGAGCGCAACGAGGTCACCGAGGTTGGCGATGCGTCGCATCTGCGTATCG<br>ACGGCGTGTTCGTGGTGGACGGTCAGCCGTGCGGCCGCTTCTCCACCAGCGCCTTGTCCCTG<br>AGTGCCGCCTTGCAGGAAGCATGGGCACAGCGCGCGATCTGAGCGCCGCGTGCCCAGAGTCA<br>TTCTGAAATTTCACTGAAACCTGCCGCGGAGACAAATCCGATGAATAGCACCAGCCTGCAAACG<br>ATTCGCCTCATCGCCGTCGATCTCGACGGCCCGTTGCTGATCGACACATTCAGCCCGATCATG<br>CACAAGCTGTGCAGCGAGTATTACCGGATCGACTACACGCGCGAACTGGAGCGCAACACCTTC<br>TCGGTACCCTCGAG
afsA<br>→ del	*GGGCCC*GAGCTCTTGACAGCTAGCTCAGTCCTAGGTATAATGCTAGC**CATCGACTGACGGGCA**<br>**GCCT**GTTTTAGAGCTAGAAATAGCAAGTTAAAATAAGGCTAGTCCGTTATCAACTTGAAAAAGTG<br>GCACCGAGTCGGTGCTTTTTTTGAATTCTCTAGAGCATCGAGCAGACCGTCGCCGCGAGCGCG<br>CCGACCAACGACGGCGAGCCGCATGGCGAGTCGGCCGATTCGCCGGCCGTCGCGCCGCCGG<br>AGCCGGAAGCGGAGCACGGCCGTCGGCGCCTCTACGCGATTCCCGCCACGACCGGCGCGCA<br>GACGCAGCCTGTCGAGCCGCCGCTGCGCCGCGATCCGCGCAACCAGTTGTGCGAGACGCTGC<br>GCATCGCGTCGGTCGAGCCGGTGCGGCTCGACGCCAAGCTGGTGGTGGACGAATCGCACGC<br>GTTCTTCTTCGACCATCCGCTCGACCATGTGTCCGGCCTGCATCTGGGCGCGGCGATGAGCGA<br>GGCGGTGCAGGCGGCCCATCTGCATCGGCACGGCCTGCCGGCCGACACCGCGCTGTTCGTAT<br>CGCAAGTGCGGCTGCACTTCGTCGATCTGTGCCGCAAGGAGCCCGGCGCGACGATCCATGTG<br>AGCGCCGCCGCCGGCGCATCCGACACGTATCAGGCGCAGTGAGCGCCGCGTGCCCAGAGTC<br>ATTCTGAAATTTCACTGAAACCTGCCGCGGAGACAAATCCGATGAATAGCACCAGCCTGCAAAC<br>GATTCGCCTCATCGCCGTCGATCTCGACGGCCCGTTGCTGATCGACACATTCAGCCCGATCAT<br>GCACAAGCTGTGCAGCGAGTATTACCGGATCGACTACACGCGCGAACTGGAGCGCAACACCTT<br>CTCGCGCTCGCGCGCCGAGGTGGTGGACTACCTTCGCCGGCAGATCGGCGAGCAGATGAGCG<br>AGACCGAACGCAAGCAGAGCGACGAGGAGAGCATCGCGAGCTATTTCCGCTATCGCGACGAAT<br>ACATGCGCGGCCATCCGCACGGCATGAAGCCCGAGGTGCCCGCCTTCCTCGATCTGCTGACC<br>TCGCTCGGCGTGACGGTGATCTGCTACGGCGGCCTCGACGAGGACTACATGCGCCGCGGGCT<br>CGGCGAACAGGCCGAGCGCTTCGCCACCTACATCTGCACGGTACC*CTCGAG*

*Psp*OMI RES; sgRNA cassette with J23119 promoter and ***afsA*-specific N20 sequence**; homologous arms F1 and F2; **mutated N20 sequence coding for E10080A and containing a *Sac*II RES**; *Xho*I RES

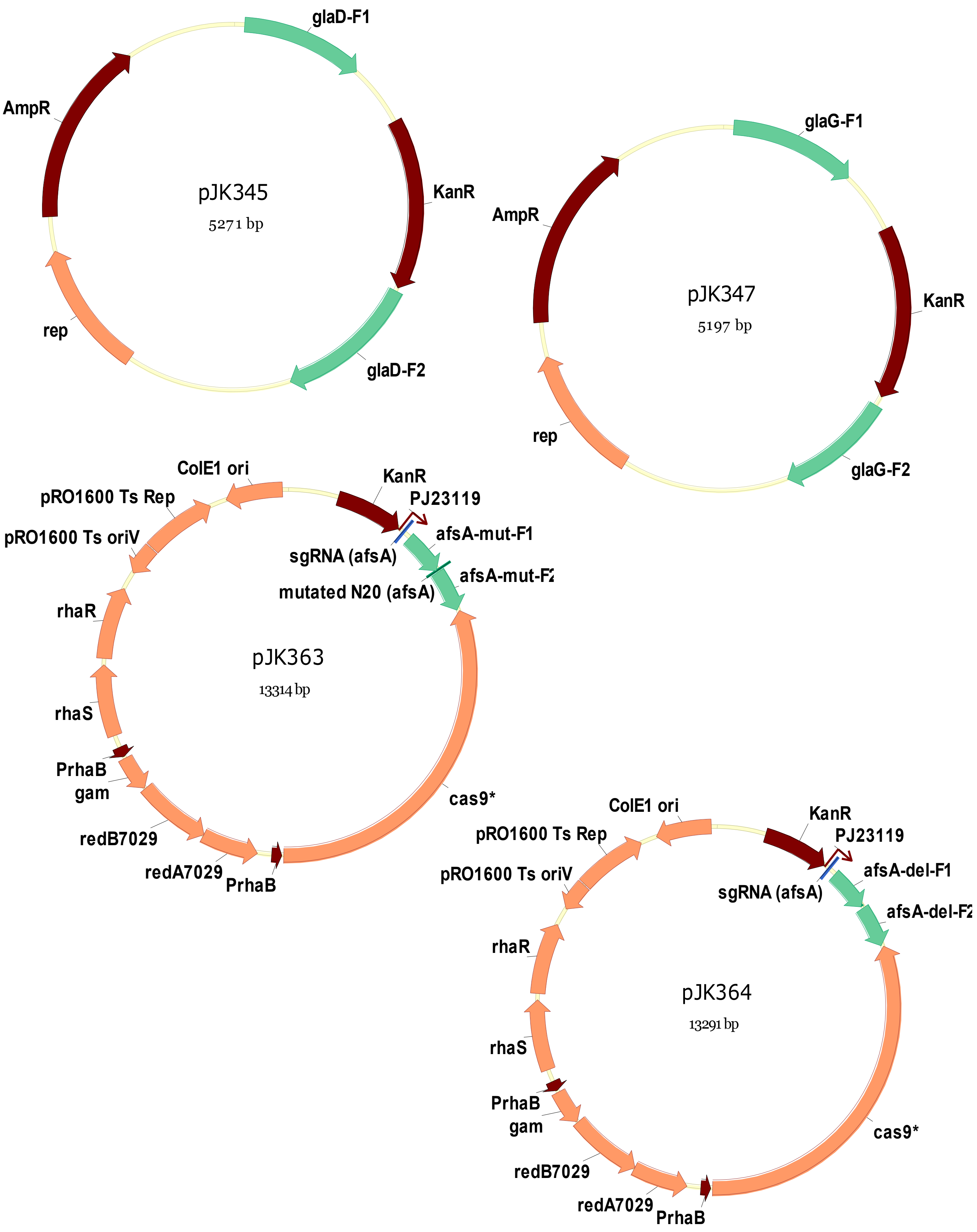

Figure S5. Maps of plasmids used for genome editing.

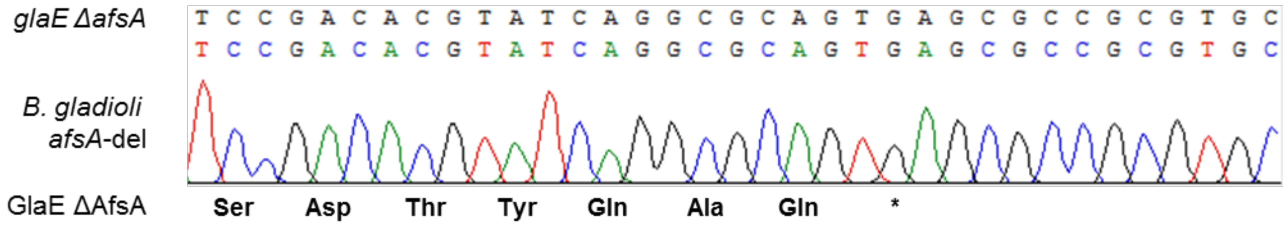

Figure S6. DNA sequence chromatogram verifying the precise deletion of the *afsA* domain region in *B. gladioli afsA*-del. The planned DNA sequence is given at the top. The corresponding amino acid sequence is given at the bottom.

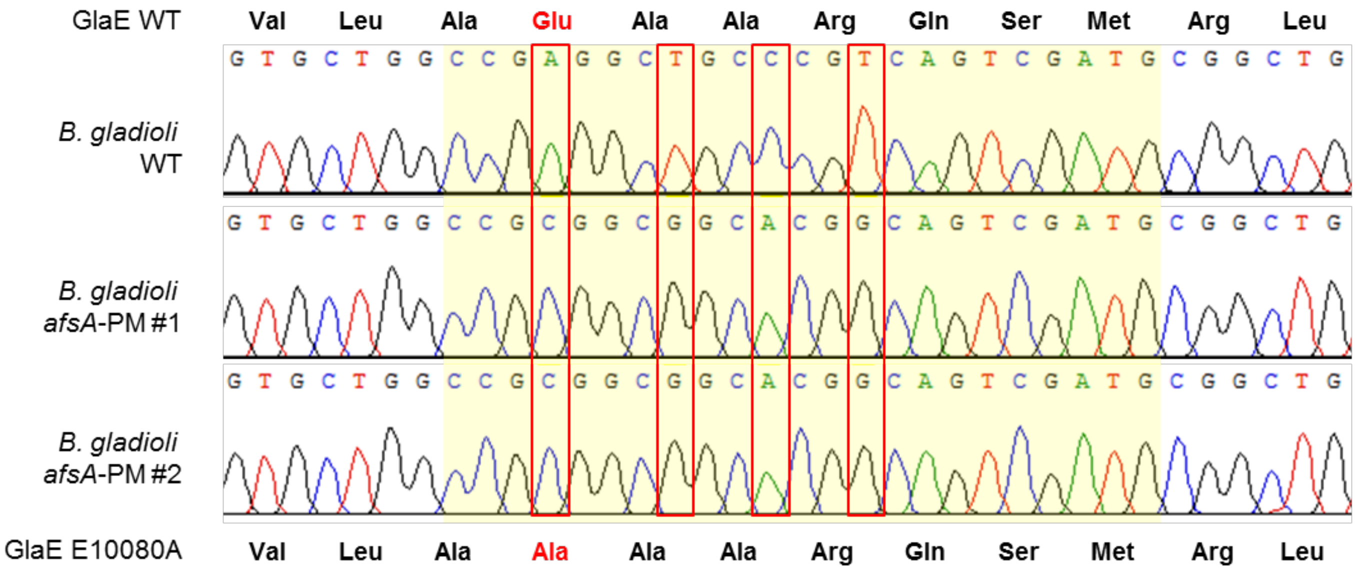

Figure S7. Comparison of DNA sequence chromatograms verifying the precise mutation of the *afsA* domain region in two obtained *B. gladioli afsA*-PM mutants. The original respectively mutated N20-PAM sequence (reverse complement) is highlighted by the yellow background; inserted point mutations are indicated by the red boxes. The corresponding amino acid sequences are given at the top and the bottom, respectively.

Off-target analysis in the *B. gladioli afsA*-del mutant

The three most prpbable gene regions for off-target effects were chosen by a BLAST search in combination with the presence of a PAM sequence (NGG) (Table S7). They were amplified and sequenced (Genewiz) from the *B. gladioli* WT and the *afsA*-deletion mutant using the primers listed in Table S5 and the KAPA2G Robust HotStart ReadyMix PCR kit (Sigma-Aldrich). Sequence alignment showed no discrepancy in any of the targets (Figure S8) indicating that there is only little risk for unwanted gene editing elsewhere in the genome. In addition, no significant difference in growth behavior could be observed (Figure S10).

Furthermore, the *B. gladioli afsA*-PM mutant that was generated using the identical sgRNA as for the *afsA*-del mutant shows a comparable metabolic profile as the WT strain (Figure S9). This indicates that at least none of the BGC of detectable metabolites was significantly altered.

Table S7. Sequences of the target and the most likely off-targets for the *afsA*-guide RNA. Marked in bold are absolute identical bases, underlined are identical but shifted bases.

(off-)target	N20-sequence	PAM
***afsA* region**	**CATCGACTGACGGGCAGCCT**	CGG
**ctg1 CDS_652**	GGCGGC**CTGACGGGCAGC**GT	CGG
**ctg1 CDS_2223**	**C**CCTCGATCT**CGGGCAGCCT**	GGG
**ctg2 CDS_2766**	GGCGCC**CTGACGGGC**G**GCCT**	CGG

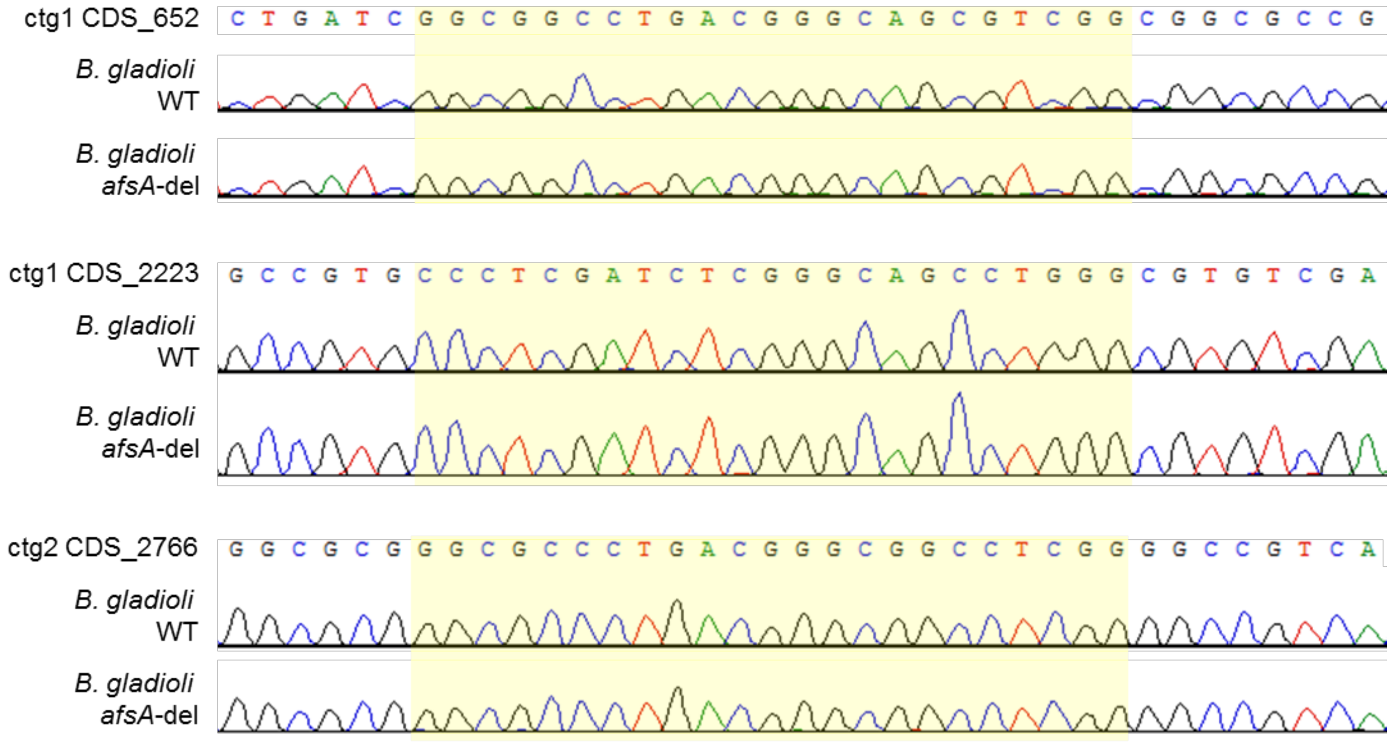

Figure S8. DNA sequence chromatograms verifying the unaltered gene sequence of the most-likely off-target regions. The yellow background highlights the N20-PAM sequence.

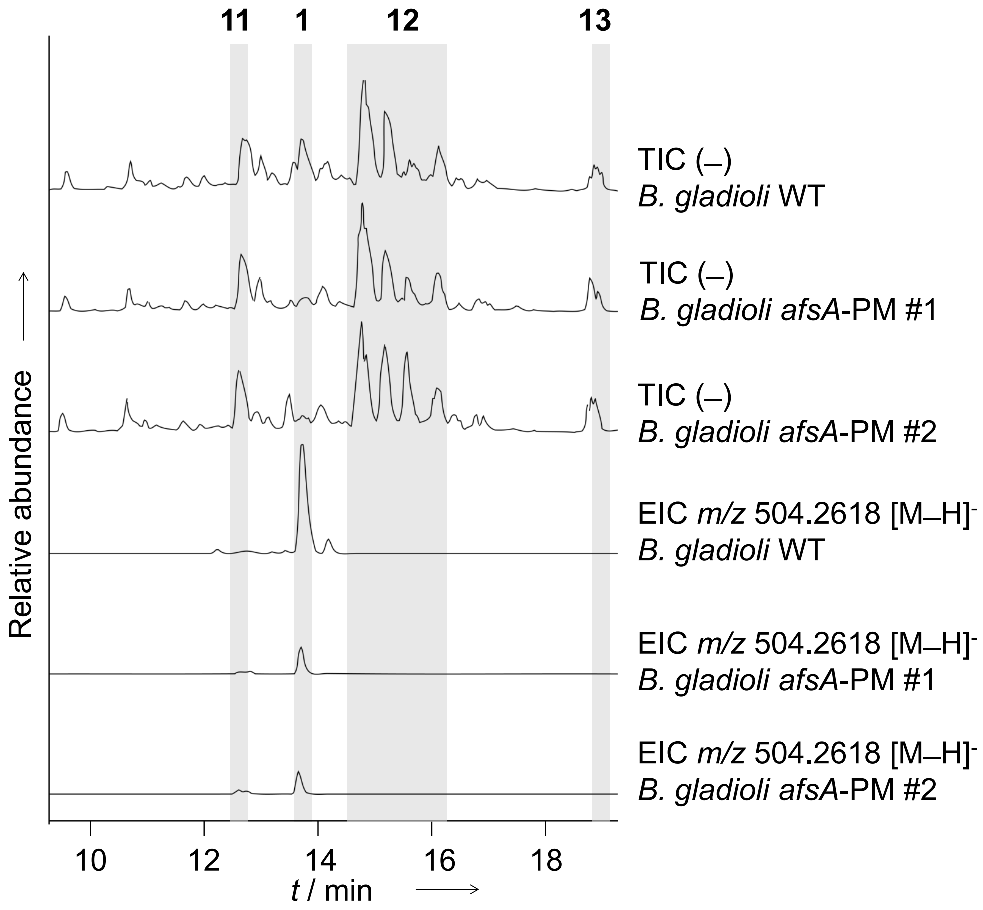

Figure S9. Metabolic profiles of *B. gladioli* HKI0739 wild type and *afsA*-PM as total ion chromatogram (TIC) and extracted ion chromatogram (EIC). Gladiofungin A (**1**), sinapigladioside (**11**), lagriene (**12**) complex, icosalide (**13**). Abundance adjusted for TIC and EIC separately.

Transcriptome analysis of the *B. gladioli afsA*-del mutant

To determine strong polar effects of the CRISPR/Cas generated gene region deletion on the transcription of the adjacent downstream genes (*glaF* and *glaG*), a qualitative RT-PCR was performed. Therefore, the *B. gladioli* WT and the *B. gladioli afsA*-del mutant were grown in PDB for 32 h at 30 °C, 120 rpm (Figure S10). Samples were taken during late exponential (OD_{600} of 0.8–1.0; 10 mL), stationary (after 8 h; OD_{600} ≈2.5; 4 mL), and late stationary growth phase (after 32 h; OD_{600} ≈10; 1.5 mL). For RNA extraction the Quick RNA Fungal Bacterial Miniprep Kit (Zymo Research) was used in combination with the TURBO DNA-*free* kit (ThermoFisher). After quantification via nanodrop approx. 2.5 µg of total RNA were treated in a 20 µL reaction using random primers and the Thermo Maxima H Minus First Strand cDNA Synthesis Kit (ThermoFisher). A non-template control (NTC) as well as a negative control for each sample (–RT) were included.

For PCR 1 µL template was added in a 10 µL reaction using the KAPA2G Robust HotStart ReadyMix PCR kit (Sigma-Aldrich). All samples were analyzed with primers JK560 and JK574 to determine the presence of specific *glaF* and *glaG* cDNA (PCR A). In addition, samples from stationary growth phase were also tested with primers JK556/JK575 for *glaD* transcription (PCR B)

and with primers JK725/JK728 for the presence of *afsA* specific cDNA (PCR C), respectively.For further verification, the product of PCR A was sequenced (Genewiz)

As shown in Figure S11, transcription of *glaF* and *glaG* could be proven in the WT strain as well as in the *afsA*-del mutant during all examined growth phases. As expected, both strains also did not differentiate regarding *glaD* transcription, but concerning the presence of the mRNA *afsA*-region.

These results in combination with the present gladiofungin production in the *B. gladioli afsA*-PM mutant (Figure S9) strongly support the hypothesis that the CRISPR/Cas genome editing did not lead to polar effects in the *B. gladioli* mutants.

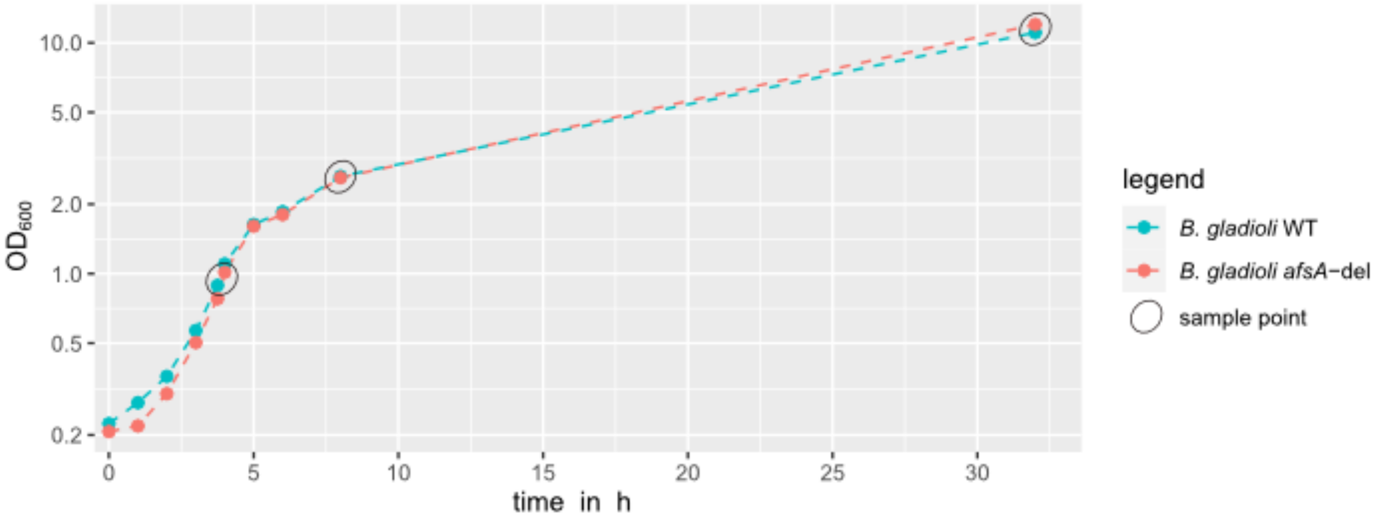

Figure S10. Growth curve of the *B. gladioli* WT and *B. gladioli afsA*-del strain for mRNA analysis.

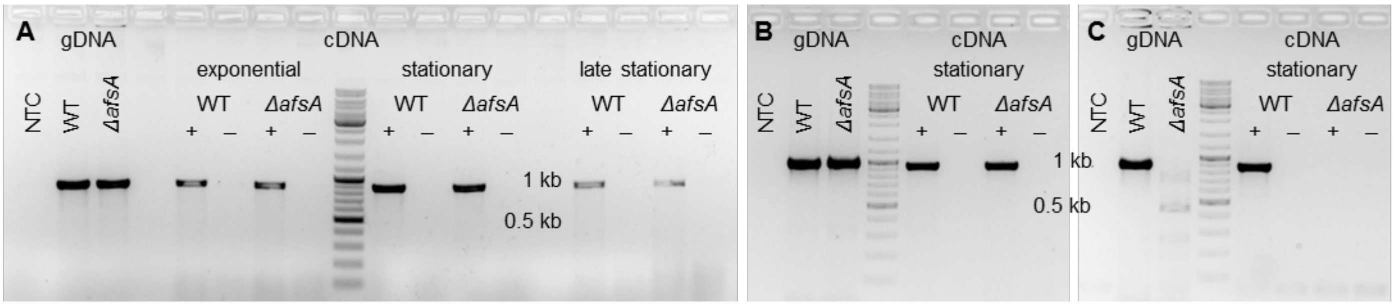

Figure S11. Qualitative RT-PCR-based verification of the expression of *glaF* and *glaG* (A), *glaD* (B), and the *afsA*-region (C) in the *B. gladioli* WT and *B. gladioli afsA*-del strain. NTC, non-template control; +, with reverse transcriptase; –, without reverse transcriptase.

Phylogenetic analysis of ketosynthase domains of gladiofungin PKS

A selection of amino acid sequences of KS domains from *trans*-AT assembly lines were used to deduce the KS specificities.[15] The amino acid sequences of the gladiofungin KS sequences were assigned according to antiSMASH analyses.[16] The multiple sequence alignment was performed using MEGA6[17] with default settings. A molecular phylogenetic tree was constructed by the Maximum Likelihood method by using IQ-tree web server (Ultrafast bootstrapping, 1000 iterations).[18] The analysis involved 204 KS amino acid sequences (Figure S12).

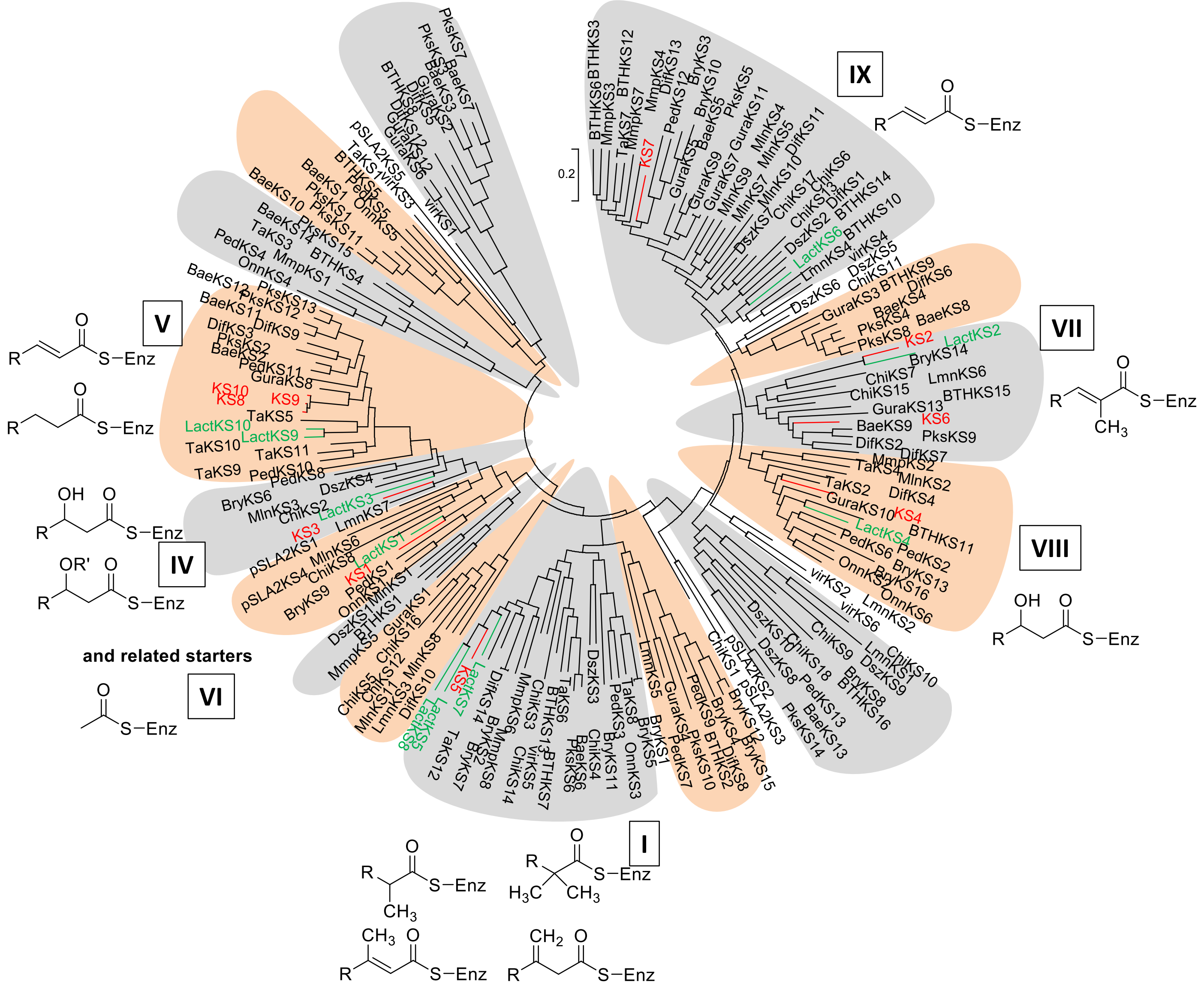

Figure S12. Maximum Likelihood tree for ketosynthase specificity. Coloration was used to emphasize the clade borders. KS (in red), Gla KS sequences labeled according to module numbers. LactKS (in green), KS sequences taken from lactimidomycin PKS.

Determining the ketoreductase specificities

The ketoreductase (KR) specificity of the domain in module 3 of the gladiofungin assembly line was predicted based on the method by Caffrey,[19] correlating the conserved HXXXXXXD motif with a D-configuration of the β-hydroxy group. KR domain amino acid sequences were aligned using Mega6[17] and a Maximum Likelihood Tree was constructed by IQ-tree web server.[18] Based on the diagnostic amino acid sequence (HAAGTLRD), the configuration at C-7 was predicted to be *R*.

The stereochemistry of C-10 attached to the methyl group in **6** was determined by using the amino acid sequence of the associated module 5 KR according to previous studies[20] (https://akitsche.shinyapps.io/profileHMM_App/). The methyl group was predicted with a ScoreDiff value of –108.83 (and an *a priori* known D-form of the secondary alcohol) as the L-form.

General analytical methods

HR-ESI-LC/MS: Exactive Orbitrap High Performance Benchtop LC-MS (Thermo Fisher Scientific) with an electron spray ion source and an Accela HPLC System, C18 column (Betasil C18, 5 µm, 150 × 2.1 mm, Thermo Fisher Scientific), solvents: acetonitrile and water (both supplemented with 0.1% formic acid (*v/v*)), flow rate: 0.2 mL min^{-1}; program: hold 1 min at 5% acetonitrile, 1–16 min 5–98% acetonitrile, hold 3 min 98% acetonitrile, 19–20 min 98% to 5% acetonitrile, hold 3 min at 5% acetonitrile.

MS/MS measurements: QExactive Orbitrap High Performance Benchtop LC/MS (Thermo Fisher Scientific) with an electron spray ion source and an Accela HPLC System, C18 column (Accucore C18, 2.6 µm, 100 × 2.1 mm, Thermo Fisher Scientific) and the following solvent system: acetonitrile and water (both supplemented with 0.1% formic acid (v/v)) at a flow rate of 0.2 mL min^{-1}; gradient: 0–10 min 5–98% acetonitrile, hold 4 min 98% acetonitrile, 14.0–14.1 min 98% to 5% acetonitrile, hold 6 min at 5% acetonitrile.

NMR: Bruker 600 MHz Avance III Ultra Shield (Bruker) and signals were referenced to the residual solvent signal at 1.94 ppm (^1H) and 118.69 ppm (^{13}C) of acetonitrile-d_3. ^1H 600 MHz, ^{13}C 150 MHz.

Isolation of gladiofungins

A Sephadex LH-20 column was used for fractionations of the methanol extract with 100% methanol as an eluent. Final purification took place by semi-preparative reversed-phase HPLC: Shimadzu LC-8A HPLC system with photo diode array, Phenomonex Synergi column 4 µ Fusion-RP 80 Å 250 × 10 mm, at a flow rate of 5 mL min^{-1}, UV detection at 190 nm. Solvents: 83% acetonitrile and H_2O with 0.01% trifluoroacetic acid. Gradient: 41.5% to 83% acetonitrile in 25 min. The collected fraction was concentrated under reduced pressure until only the water phase remained. The remaining mixture was frozen at –20 °C and lyophilized overnight.

Labeling experiments

B. gladioli HKI0739 was inoculated on a NAG agar plate and grown at 30 °C. A bacterial colony was transferred into 2 mL MGY+M9 medium and grown at 30 °C and 110 rpm for 24 h. The resulting cultured cells were inoculated into 20 mL of MGY+M9 medium with an OD_{600} of 0.1 and grown under the same conditions for 24 h. $^{13}C_3$-labeled glycerol (500 mg) were added to the growing culture. After additional 21 h of growing (under the same conditions) the culture was extracted three times with an equal volume of ethyl acetate. The obtaining organic phases were combined and concentrated under the reduced pressure. The residue was dissolved in a small volume of acetonitrile. Final purification was achieved by semi-preparative reversed-phase HPLC as described above.

Determination of bioactivity

Antibacterial and antifungal profiling was carried out in an agar diffusion assays as previously described (Table S8).[21] In addition, gladiofungin A (dissolved in methanol) was tested in different concentrations in an agar diffusion assay against the entomopathogenic *Purpureocillium lilacinum* (Figure S13 and Table S9). Cytotoxic and antiproliferative activities were tested as described previously (Table S10).[22] Here, all substances were dissolved in DMSO.

Table S8. Inhibitory effects of gladiofungin A against several bacterial and fungal strains.

**Strain**	**Zone of inhibiton in mm**		
	Gladiofungin A (1 mg mL^{-1}; 1.98 mM)	Ciprofloxacin (5 µg mL^{-1}; 3 mM) / Amphotericin B (10 mg mL^{-1}; 1.1 mM)	Methanol
Bacillus subtilis 6633 B1	12	29	0
Pseudomonas aeruginosa K799/61 B7	12p	26	0
Staphylococcus aureus 134/94 R9 (MRSA)	14p	0	0
Enterococcus faecalis 1528 R10 (VRE)	13p	16	0
Mycobacterium vaccae 10670 M4	0	21p	0
Sporobolomyces salmonicolor 549 H4	32p	18p	10
Candida albicans H8	0	20	0
Penicillium notatum JP36 P1	31p	19p	10

p – partial inhibition

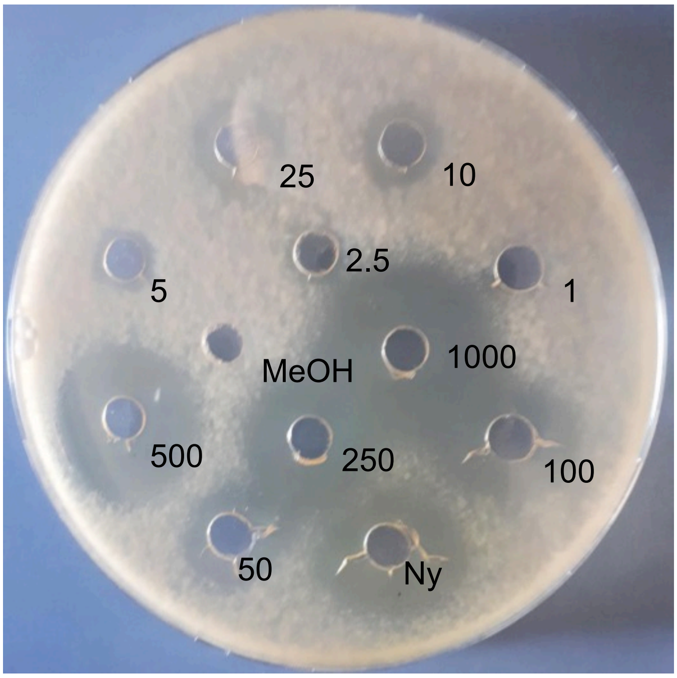

Figure S13. Agar diffusion assay for *Purpureocillium lilacinum*. Ny – Nystatin 800 µg mL^{-1}**.**

Table S9. Agar diffusion assay against *Purpureocillium lilacinum* with inhibition zone. Test hole 9 mm.

	**Concentration [µg mL^{-1}]**	**Inhibition zone**
Gladiofungin A (**1**)	1000	23
	500	20
	250	19
	100	17
	50	14
	25	13
	10	12
	5	10.5
	2.5	10
	1	-
Nystatin (800 µg mL^{-1})		18
Methanol		-

Table S10. Cytotoxic and antiproliferative profiling of gladiofungin A.

**Cell line**	**GI_{50} [µg mL^{-1}]**	**GI_{50} [µM]**	**CC_{50} [µg mL^{-1}]**	**CC_{50} [µM]**
HUVEC	25.8	51.1	-	-
K-562	23.3	46.14	-	-
THP-1	3.6	7.1	-	-
HEK-293	5.6	11.1	-	-
HeLa	-	-	30.6	60.6

MS/MS spectra

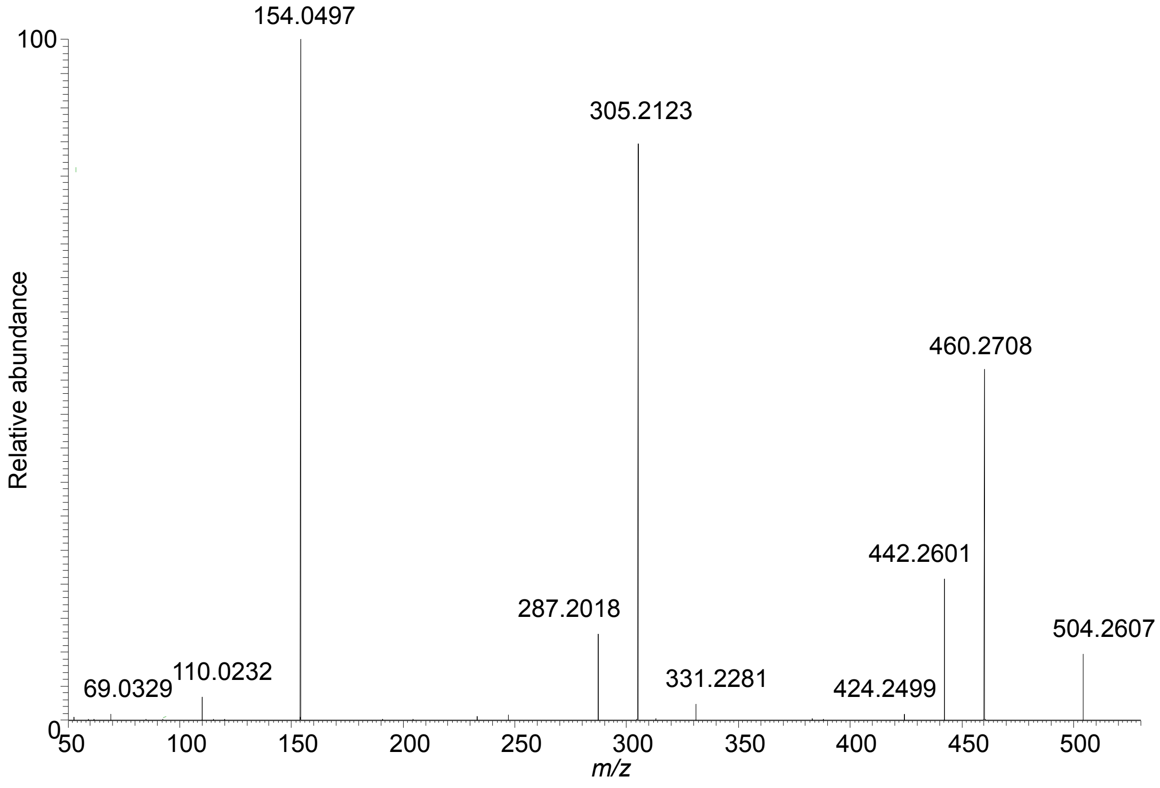

Figure S14. MS/MS fragmentation pattern of gladiofungin A (1) (*m/z* 504.2604 [M–H]⁻).

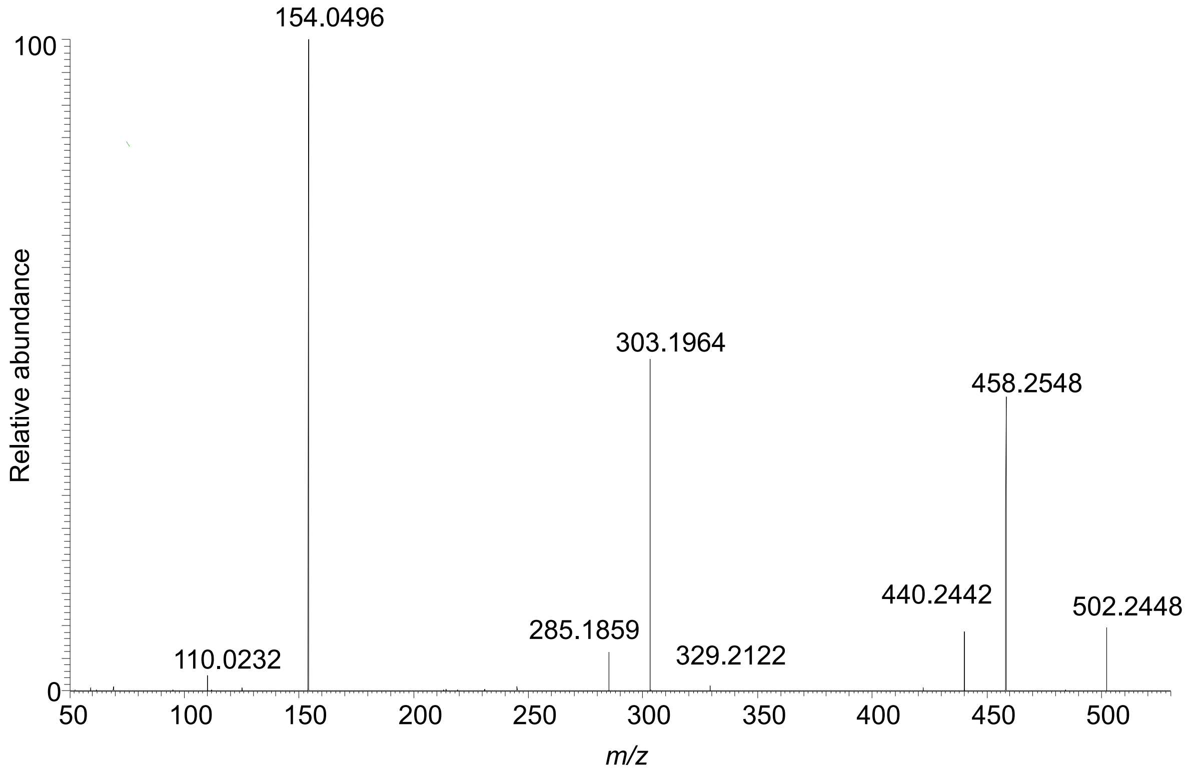

Figure S15. MS/MS fragmentation pattern of gladiofungin B (2) (*m/z* 502.2448 [M–H]⁻).

NMR tables

Table S11. NMR shifts of gladiofungin A (1).

Position	δ_C [ppm]	δ_H [ppm]; Signal (*J* [Hz])
1	174.1[a]	-
2	39.3[b]	2.60; 2 H m
3	28.5	*
4	38.2[b]	2.64; 2 H m
5	174.2[a]	-
6	42.7	1.35; 1 H m<br>1.45; 1 H m
7	65.9	4.05; 1 H m
8	49.1	2.57; 2 H m
9	212.7	-
10	51.6	3.18; 1 H quin (*7.1*)
11 (-CH_3)	16.6	1.06; 3 H d (*6.8*)
12	130.4	5.36; 1 H m
13	135.1	5.60; 1 H dt (*15.4; 6.6*)
14	33.5	2.00; 2 H q (*6.6*)
15	30.4	1.35; 2 H m
16	30.0[c]	1.28; 2 H m
17	30.1[c]	1.28; 2 H m
18	30.2[c]	1.28; 2 H m
19	30.3[c]	1.28; 2 H m
20	24.5	1.56; 2 H m
21	43.5	2.85; 2 H td (*7.5*; *1.4*)
22	199.2	-
23	128.0	-
24	169.7	-
25	98.9	5.89; 1 H s
26	172.3	-
27(-CH_3)	14.0	2.24; 3 H s

[a]–[c] interchangeable signals * overlay with solvent signal

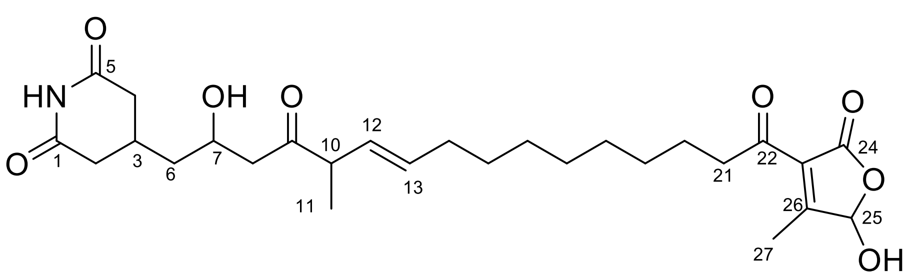

Table S12. ^1H NMR shifts of gladiofungin B (2).

Position	***δ*$_H$ [ppm]; Signal (*J* [Hz])**
1	-
2	2.61; 2 H m
3	*
4	2.64; 2 H m
5	-
6	1.38; 1 H m<br>1.46; 1 H m
7	4.06; 1 H m
8	2.58; 2 H m
9	-
10	3.25; 1 H quin (*7.3*)
11 (-CH_3)	1.11; 3 H d (*6.8*)
12	5.70; 1 H dt (*14.6; 7.4*)
13	6.16; 1 H dd (*15.0; 10.3*)
14**	6.07; 1 H dd (*15.4; 10.1*)
15**	5.51; 1 H dd (*15.4*; *8.8*)
16	2.08; 2 H m
17	1.38; 2 H m
18	1.29; 2 H m
19	1.29; 2 H m
20	1.56; 2 H m
21	2.85; 2 H td (*7.5*; *1.8*)
22	-
23	-
24	-
25	5.90; 1 H s
26	-
27 (-CH_3)	2.25; 3 H s

** CH=CH instead of CH_2-CH_2 as in **1**

Detailed structure elucidation

The structure of **1** was elucidated by 1D- and 2D-NMR. ^{13}C NMR and DEPT-135 data of **1** indicated the presence of 6 methines, 12 methylenes, 2 methyl groups, and 7 quaternary carbons, from which 5 were assigned to carbonyls. In extensive ^{1}H-^{1}H COSY analyses, three spin systems (from H-2 to H-8, from H-10 to H-16, and H-19 to H-21) were observed. The first fragment (H-2 to H-8) contains the glutarimide moiety, judging from the ^{1}H-^{13}C HMBC correlations from NH (δ 8.64 ppm)/H-2 to C-1 (δ 172.1 ppm) and from NH/H4- to C-5 (δ 172.2 ppm). The second fragment (H-10 to H-16) was connected via keto carbonyl carbon C-9 (δ 212.7 ppm) with the first fragment by the HMBC correlations between H-8/H-10 and C-9. The HMBC correlation from H-21 to the keto carbonyl carbon C-22 (δ 199.2 ppm) connects with the third fragment (C-19 to H-21). A quaternary carbon rich heterocyclic moiety, which is adjacent to C-22, was deduced as tri-substituted butenolide by four HMBC correlations from singlet methyl protons 27-CH_3 (δ 2.24 ppm) to C-22/C-23 (δ 128.0 ppm)/C-25 (δ 98.9 ppm) and from singlet hemiacetal methine proton H-25 (δ 5.89 ppm) to C-24 (δ 169.7 ppm). To increase ^{13}C NMR signals in this heterocyclic moiety, **1** was ^{13}C-enriched by isotope labeling using ubi-$^{13}C_3$-glycerol. The ADEQUATE correlations from 27-CH_3/H-25 to C-26 and the INADEQUATE correlation between C-23 and C-24, respectively, obtained from ^{13}C-enriched **1** supported to elucidate the five-membered ring structure (Figure 3B). Finally, the observed INADEQUATE correlations from C16 to C19 completed the structure of **1**. The *E*-configuration of the double bond C-12/C-13 (δ_H 5.36 and 5.60 ppm) was assigned based on the proton coupling constant $J_{12–13}$ 15.5 Hz.

NMR spectra

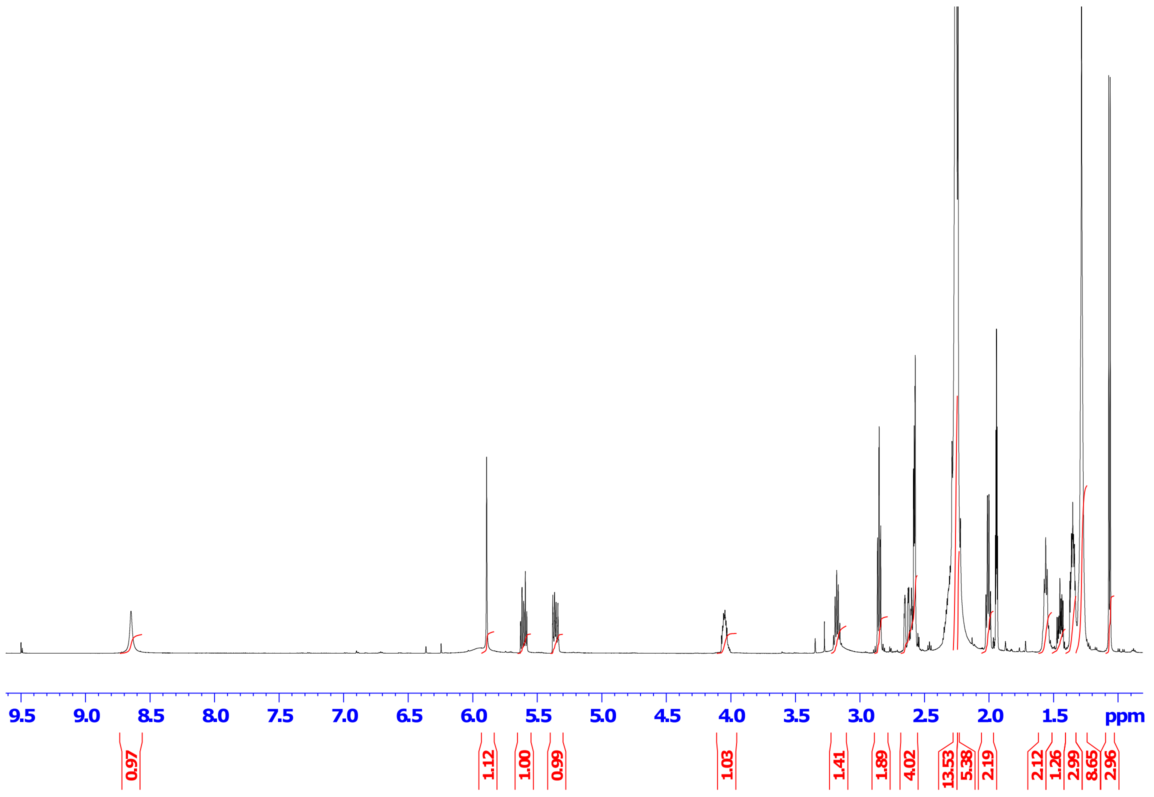

Figure S16. ^{1}H NMR spectrum of gladiofungin A (1).

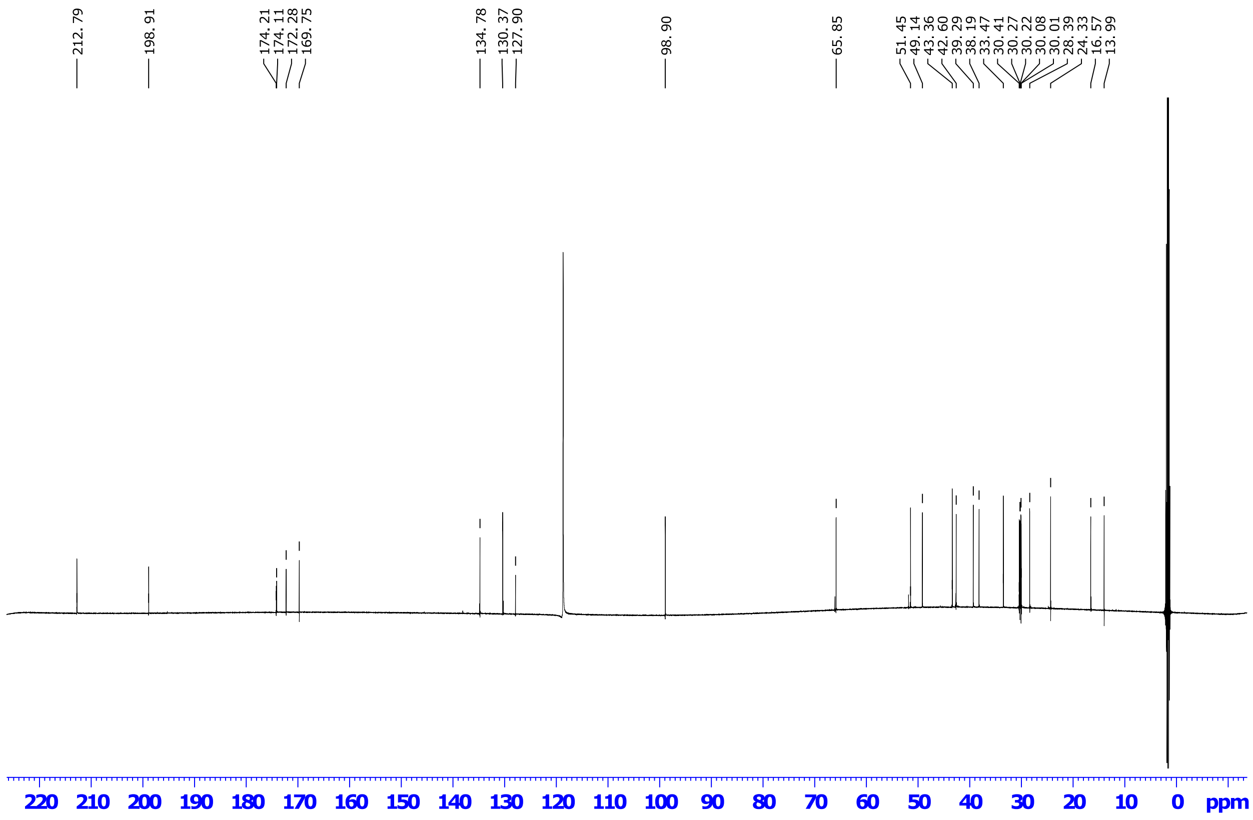

Figure S17. ^{13}C NMR spectrum of gladiofungin A (1).

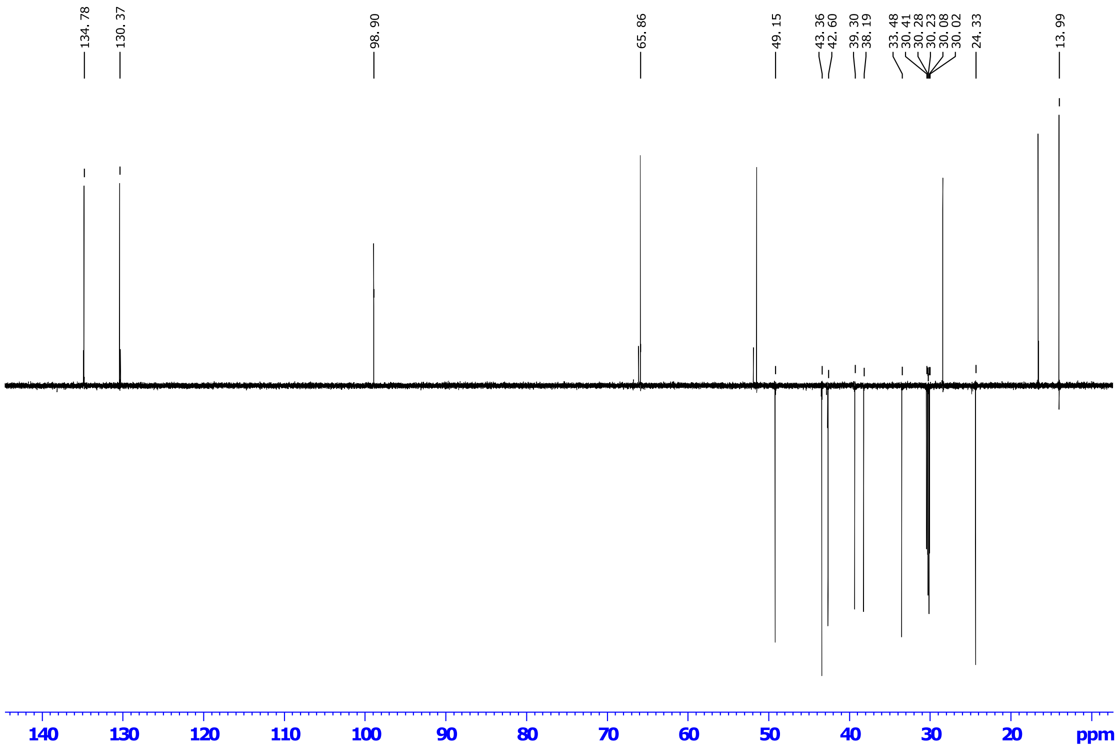

Figure S18. DEPT135 NMR spectrum of gladiofungin A (1).

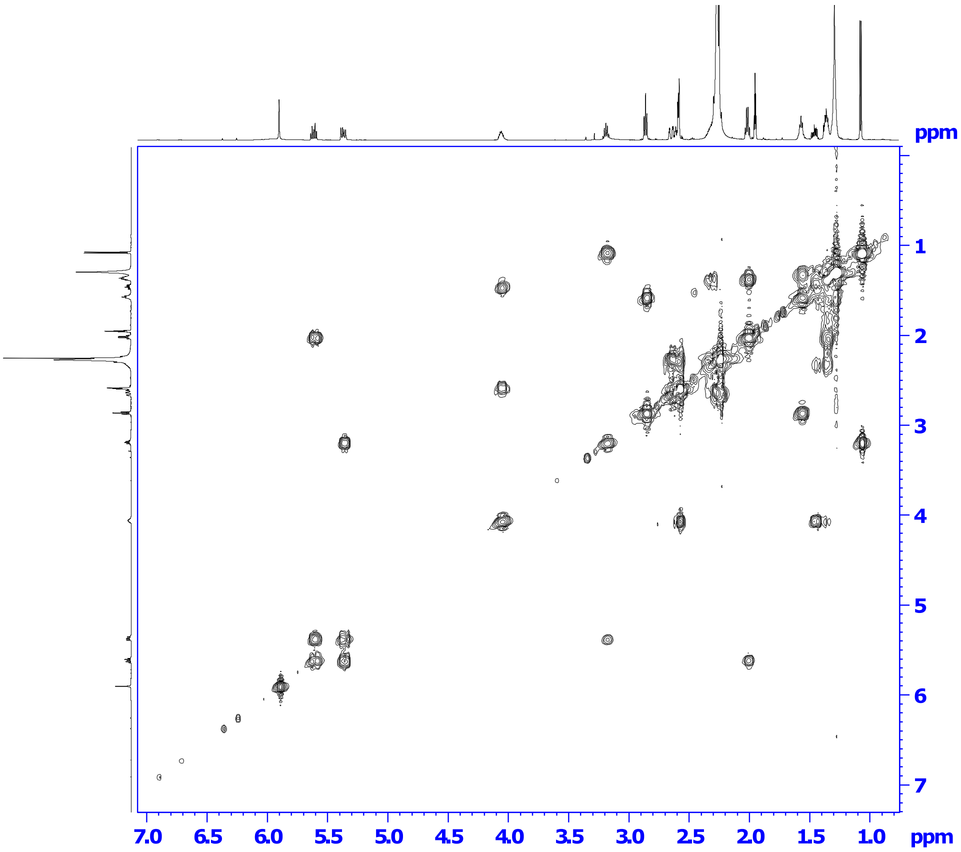

Figure S19. ^{1}H-^{1}H COSY NMR spectrum of gladiofungin A (1).

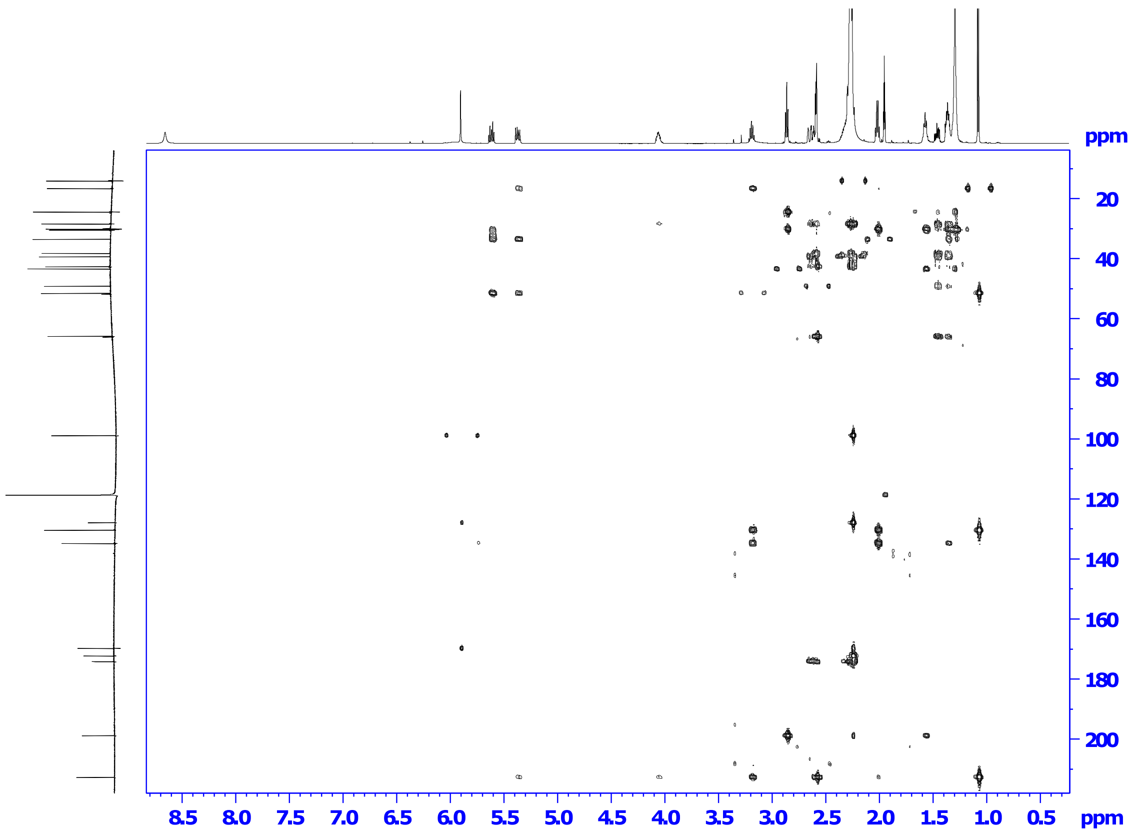

Figure S20. ^{1}H-^{13}C **HMBC NMR spectrum of gladiofungin A (1).**

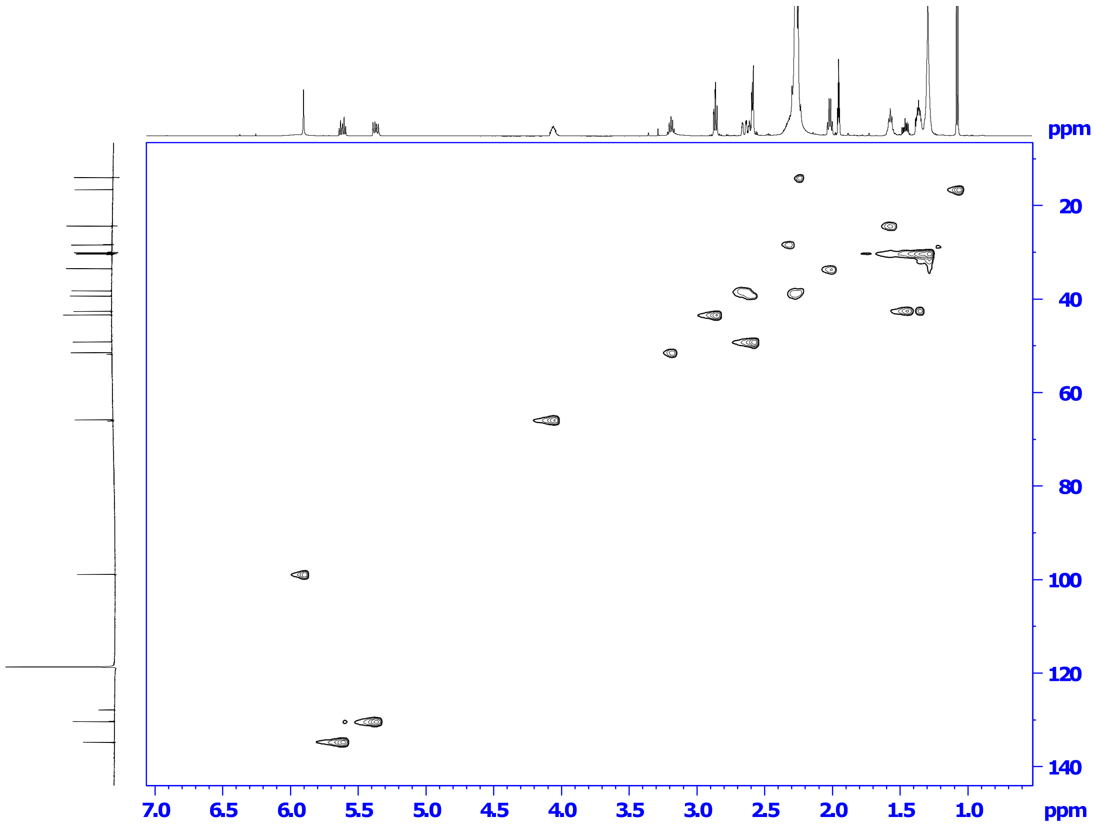

Figure S21. ^{1}H-^{13}C **HSQC NMR spectrum of gladiofungin A (1).**

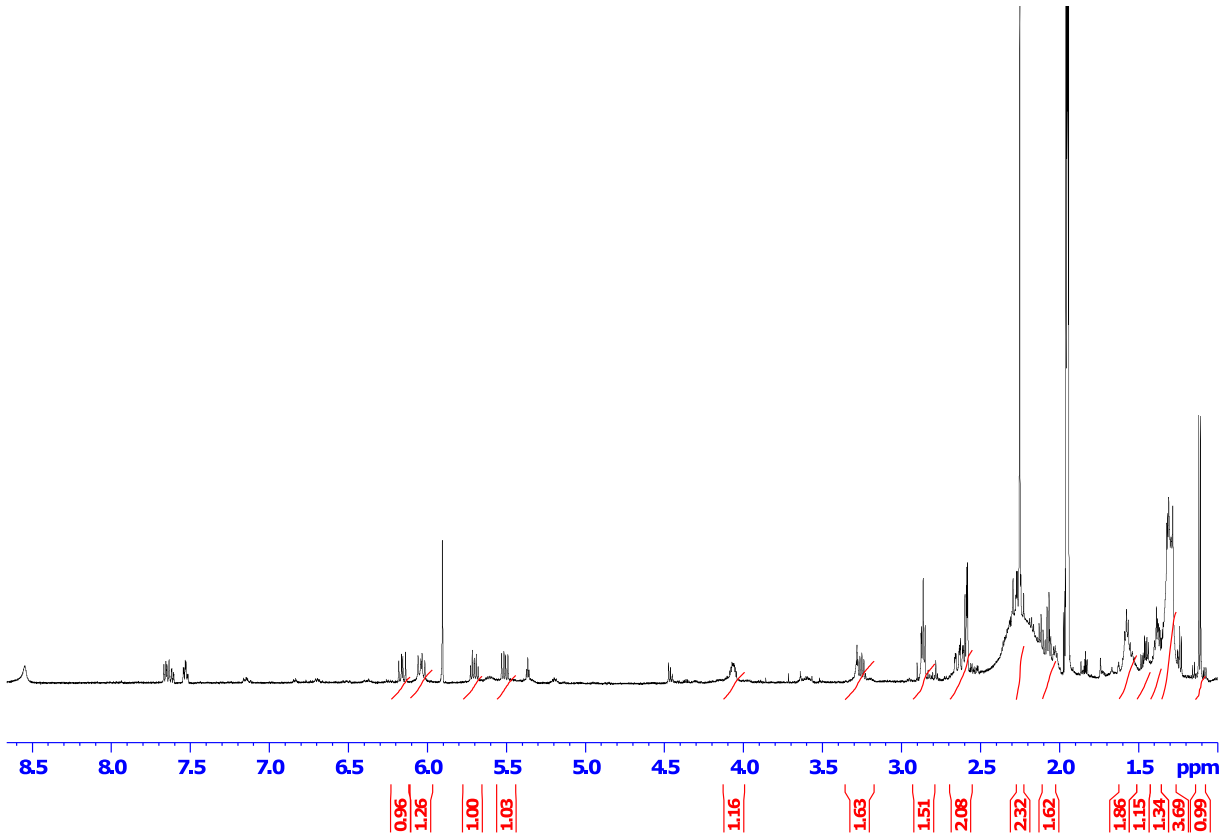

Figure S22. ^{1}H NMR spectrum of gladiofungin B (2).

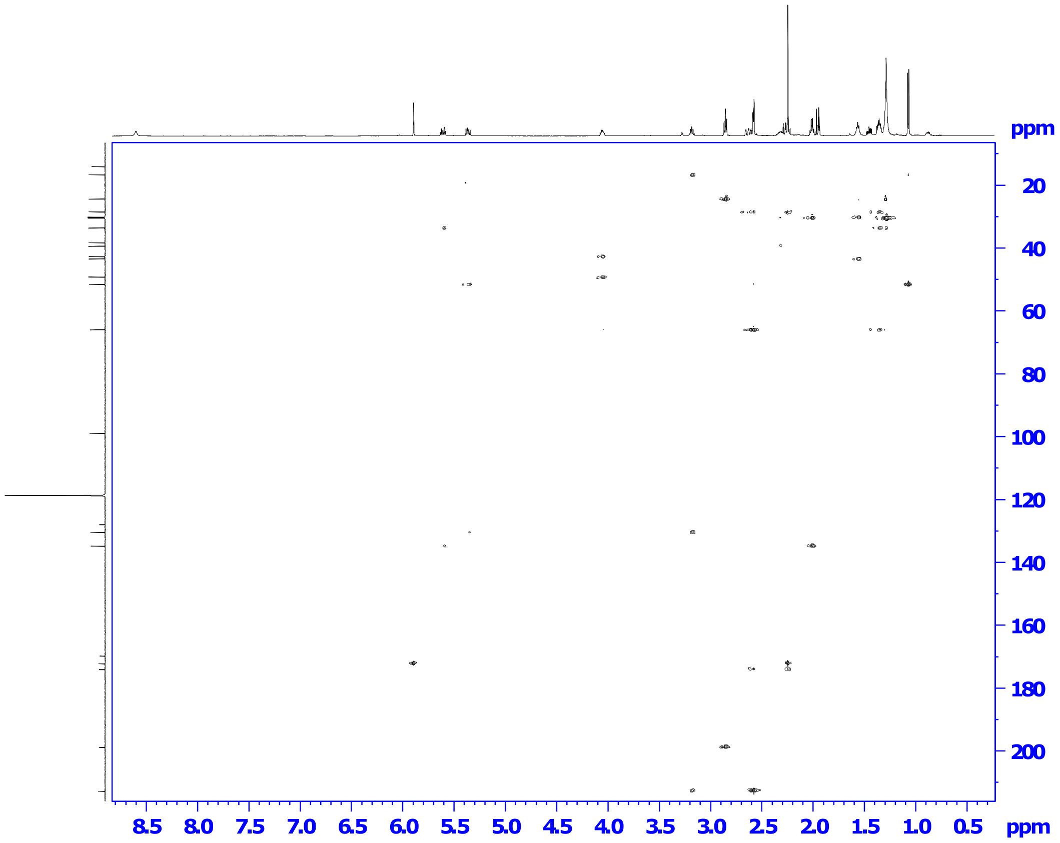

Figure S23. ADEQUATE NMR spectrum of gladiofungin A (1).

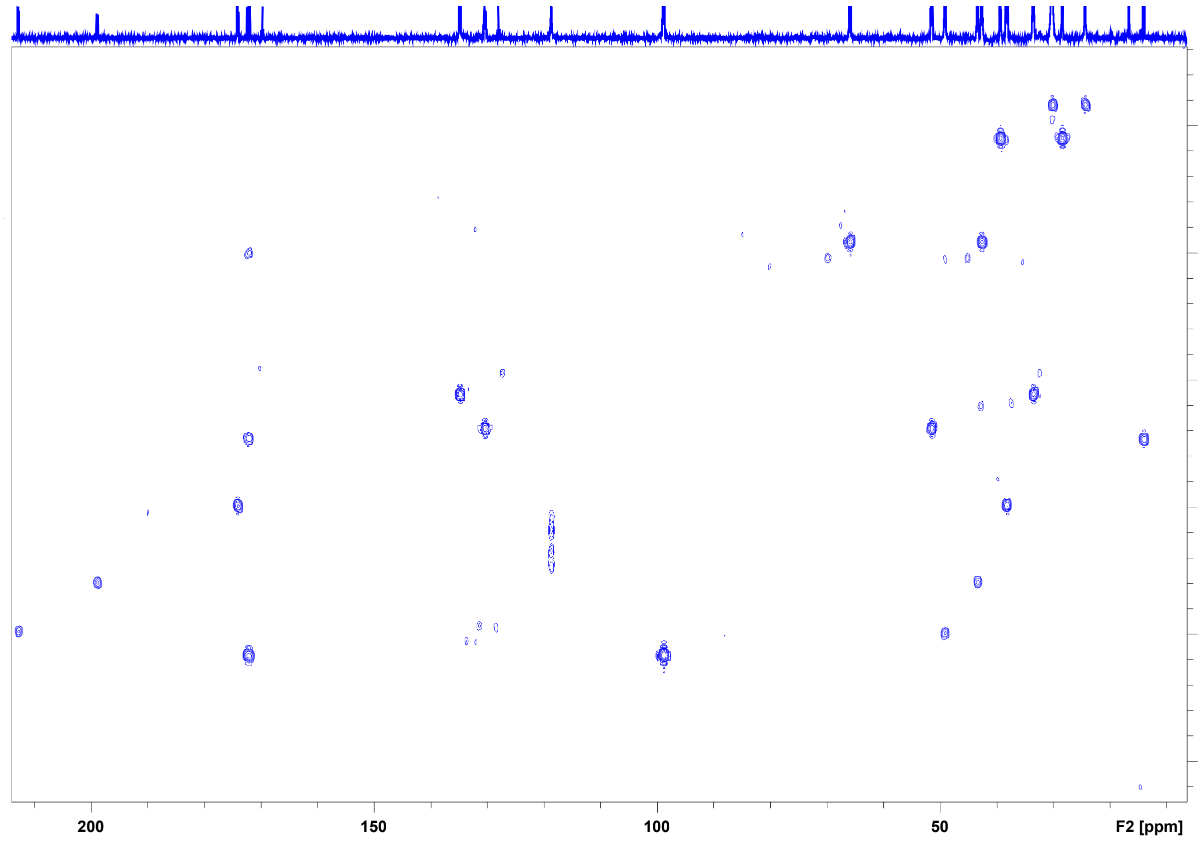

Figure S24. INADEQUATE NMR spectrum of ^{13}C-labeled gladiofungin A (1).

Supplemental References

[1] K. Blin, S. Shaw, K. Steinke, R. Villebro, N. Ziemert, S. Y. Lee, M. H. Medema, T. Weber, *Nucleic Acids Res.* **2019**, *47*, W81-W87.

[2] B. O. Bachmann, J. Ravel, *Methods Enzymol.* **2009**, *458*, 181-217.

[3] L. Zimmermann, A. Stephens, S.-Z. Nam, D. Rau, J. Kübler, M. Lozajic, F. Gabler, J. Söding, A. N. Lupas, V. Alva, *J. Mol. Biol.* **2018**, *430*, 2237-2243.

[4] J. A. Gerlt, J. T. Bouvier, D. B. Davidson, H. J. Imker, B. Sadkhin, D. R. Slater, K. L. Whalen, *Biochim. Biophys. Acta. Proteins Proteom.* **2015**, *1854*, 1019-1037.

[5] J.-W. Seo, M. Ma, T. Kwong, J. Ju, S.-K. Lim, H. Jiang, J. R. Lohman, C. Yang, J. Cleveland, E. Zazopoulos, *Biochemistry* **2014**, *53*, 7854-7865.

[6] B. Wang, Y. Song, M. Luo, Q. Chen, J. Ma, H. Huang, J. Ju, *Org. Lett.* **2013**, *15*, 1278-1281.

[7] M. Yin, Y. Yan, J. R. Lohman, S.-X. Huang, M. Ma, G.-R. Zhao, L.-H. Xu, W. Xiang, B. Shen, *Org. Lett.* **2014**, *16*, 3072-3075.

[8] K. Ishida, T. Lincke, C. Hertweck, *Angew. Chem. Int. Ed.* **2012**, *51*, 5470-5474.

[9] X. Wang, H. Zhou, H. Chen, X. Jing, W. Zheng, R. Li, T. Sun, J. Liu, J. Fu, L. Huo, *Proc. Natl. Acad. Sci. U. S. A.* **2018**, *115*, E4255-E4263.

[10] A. M. Hogan, A. S. M. Z. Rahman, T. J. Lightly, S. T. Cardona, *ACS Synth. Biol.* **2019**, *8*, 2372-2384.

[11] K.-H. Choi, T. Mima, Y. Casart, D. Rholl, A. Kumar, I. R. Beacham, H. P. Schweizer, *Appl. Environ. Microbiol.* **2008**, *74*, 1064-1075.

[12] B. Doublet, G. Douard, H. Targant, D. Meunier, J.-Y. Madec, A. Cloeckaert, *J. Microbiol. Methods* **2008**, *75*, 359-361.

[13] L. V. Flórez, K. Scherlach, P. Gaube, C. Ross, E. Sitte, C. Hermes, A. Rodrigues, C. Hertweck, M. Kaltenpoth, *Nat. Commun.* **2017**, *8*, 15172.

[14] S. T. Cardona, M. A. Valvano, *Plasmid* **2005**, *54*, 219-228.

[15] T. Nguyen, K. Ishida, H. Jenke-Kodama, E. Dittmann, C. Gurgui, T. Hochmuth, S. Taudien, M. Platzer, C. Hertweck, J. Piel, *Nat. Biotech.* **2008**, *26*, 225.

[16] K. Blin, T. Wolf, M. G. Chevrette, X. Lu, C. J. Schwalen, S. A. Kautsar, H. G. Suarez Duran, E. L. De Los Santos, H. U. Kim, M. Nave, *Nucleic Acids Res.* **2017**, *45*, W36-W41.

[17] K. Tamura, G. Stecher, D. Peterson, A. Filipski, S. Kumar, *Mol. Biol. Evol.* **2013**, *30*, 2725-2729.

[18] J. Trifinopoulos, L.-T. Nguyen, A. von Haeseler, B. Q. Minh, *Nucleic Acids Res.* **2016**, *44*, W232-W235.

[19] P. Caffrey, *ChemBioChem* **2003**, *4*, 654-657.

[20] A. Kitsche, M. Kalesse, *ChemBioChem* **2013**, *14*, 851-861.

[21] K. Scherlach, L. P. Partida-Martinez, H.-M. Dahse, C. Hertweck, *J. Am. Chem. Soc.* **2006**, *128*, 11529-11536.

[22] R. Abdou, K. Scherlach, H.-M. Dahse, I. Sattler, C. Hertweck, *Phytochemistry* **2010**, *71*, 110-116.
